# Effects of the gut microbiota on placental angiogenesis and intrauterine growth in gnotobiotic mice

**DOI:** 10.1073/pnas.2426341122

**Published:** 2025-07-25

**Authors:** Reyan Coskun, ZeNan L. Chang, Athziri Marcial Rodríguez, Haoxin Liu, Jiye Cheng, Yael Alippe, Michael S. Diamond, Jeffrey I. Gordon

**Affiliations:** ^a^The Edison Family Center for Genome Sciences and Systems Biology, Washington University School of Medicine, St. Louis, MO 63110; ^b^The Newman Center for Gut Microbiome and Nutrition Research, Washington University School of Medicine, St. Louis, MO 63110; ^c^Department of Medicine, Washington University School of Medicine, St. Louis, MO 63110; ^d^Department of Pathology and Immunology, Washington University School of Medicine, St. Louis, MO 63110; ^e^Department of Molecular Microbiology, Washington University School of Medicine, St. Louis, MO 63110

**Keywords:** gut microbiota in pregnancy, fetal-placental development, intrauterine growth restriction, angiogenesis, gnotobiotic mice

## Abstract

Environmental causes of intrauterine growth restriction (IUGR) remain poorly characterized. Here, we compare germ-free (GF) and conventionally raised (CONV-R) mice to assess the effects of the gut microbiota on placental/fetal development at embryonic day (E)11.5 (end of placentation) and E17.5 (near term). Pregnancy- and microbiota-associated changes in gene expression occur along the gut, including those related to angiogenesis, while bacterial composition and fermentation activity remain stable. Placental weights at E11.5 and fetal weights at E17.5 are significantly reduced in GF animals. Compared to CONV-R dams, the GF maternal decidua exhibits similar vascular histomorphometric features at E11.5 and E17.5, and numbers of uterine NK-cells (effectors of vascular remodeling) at E11.5. In contrast, angiogenesis is disturbed in the GF fetal-derived placental compartment (junctional and/or labyrinth zones) at E11.5, as judged by i) increased levels of proangiogenic proteins (angiopoietin-2, FGF-2, follistatin, SDF-1, VEGF-A, VEGF-C); ii) increased levels of phos-VEGFR2 and phos-p38-MAPK yet reduction in phos-ERK1/2; and iii) reduced expression of junctional zone glycoprotein genes associated with angiogenesis and fetal growth, resulting in reduced endothelial cell density at the labyrinth zone at E17.5. Colonization of GF mice before pregnancy with cecal microbiota from CONV-R animals rescues fetal growth and altered transcriptomic, proteomic, and immunohistochemical features in the fetal GF placental compartment. Single-nucleus RNA-sequencing demonstrated increased expression of mitochondrial and ribosomal-associated oxidative stress genes in endothelial cell clusters in the GF fetal placental compartment (E11.5, E17.5), mimicking oxidative stress signatures in human IUGR. These results provide a rationale for seeking microbial targets for treating/preventing IUGR.

## Significance

Intrauterine growth restriction (IUGR) impacts ~30% of pregnancies in low- and middle-income countries, but limited treatment options are available. The placenta has distinct compartments, with different specialized cells populating the maternally derived decidua and fetal-derived junctional and labyrinth zones. The gut microbiota’s influence on placental cellular composition and function during gestation is not well defined. Comparing germ-free female mice and their normally colonized counterparts disclosed fetal and placental growth restriction accompanied by gene and protein expression shifts, notably those reflecting abnormal blood-vessel development in fetal-derived placental compartments. Providing a mouse gut microbiota to germ-free mice prior to pregnancy rescues abnormal placental vascularization and fetal growth restriction, suggesting that the maternal gut microbiota is a potential therapeutic target for treating/preventing IUGR.

The intrauterine environment is a critical determinant of healthy fetal development throughout pregnancy. Intrauterine growth restriction (IUGR) is defined as a decreased rate of growth compared to the genetic potential of a fetus at a particular gestational age (1). The incidence of “small for gestational age” (SGA), a common epidemiologic proxy for IUGR, is 10 to 15% in high-income countries (HIC) and up to 30% in low- and middle-income countries (LMICs) (2). Fetuses with IUGR are at increased risk for premature birth. Fetuses growing in a suboptimal intrauterine environment are thought to redirect nutrients to essential organs by modifying their endocrine-metabolic pathways and experience vascular modifications that induce global endothelial damage (3). This reprogramming predisposes to cardiovascular, kidney, and liver disease as well as obesity/metabolic syndrome and diabetes in adulthood (4).

IUGR has been linked to placental dysfunction. In healthy human pregnancies, uterine artery branches are converted into low resistance uteroplacental vessels to maximize maternal blood flow to the fetal vascular bed for adequate nutrient and oxygen transfer. In pregnancies with IUGR, there is evidence of high-resistance flow in these vessels (5) and oxidative and endoplasmic reticulum (ER) stress responses (6, 7). Placentas in humans with IUGR and in animal models also have decreases in amino acid transport across maternal facing syncytiotrophoblasts (8) and of lipoprotein lipase that releases free fatty acids into maternal blood (9).

Although the series of hormonal, metabolic, and immunological changes that occur in mothers to support their developing fetus have been the focus of much research, the contributions of the maternal gut microbiota, which encodes functions not represented in our *Homo sapiens* genomes, remain poorly understood (10). For example, the extent to which the microbiota undergoes a definable “normal” program of change during pregnancy, and if so, what the physiological consequences of such changes might be, are unclear. Studies have characterized changes in bacterial composition in the fecal microbiota in human pregnancy, including one that demonstrated minimal shifts in gut community diversity metrics (11) and another that found a change in the representation of *Bifidobacterium* (12).

Maternal gut microbial community composition has been shown to broadly affect postnatal development in mouse models including the representation of intestinal group 3 innate lymphoid cells (ILCs) and F4/80^+^ CD11c^+^ mononuclear cells, expression of intestinal epithelial-derived antimicrobial peptides (13), and fetal brain maturation (14). Pronovost et al. noted that germ-free (GF) female mice that had either been reared in a sterile environment or conventionally raised (CONV-R) and then subjected to broad-spectrum antibiotic depletion of their gut microbiota, had reduced placental weights compared to GF dams colonized during pregnancy with a gut microbiota harvested from CONV-R mice [yielding “conventionalized” (CONV-D) animals]. The GF and the antibiotic-treated mice had reduced placental vascular volume, surface area, and branching (15).

Like humans, mice have hemochorial placentas, the most invasive type of placenta in which there is erosion/decidualization of maternal tissue allowing for a direct connection between the placenta and maternal blood supplies (16). The mouse placenta consists of three discrete zones–the labyrinth zone (LZ), the junctional zone (JZ), plus the maternally derived decidua and mesometrial lymphoid aggregate of pregnancy (MLAp) (Fig. 2*A*). The LZ is the site of maternal–fetal nutrient and oxygen exchange and consists of maternal sinusoidal-like vasculature and fetal blood vessels. The mouse LZ has three trophoblast layers separating the maternal and fetal blood supplies; the primary cell types are syncytiotrophoblasts and sinusoidal trophoblastic giant cells (S-TGCs) (17). The JZ lies directly adjacent to the LZ and has contact with maternal tissue; it consists of three main cell types – spongiotrophoblasts (SpT), glycogen cells (GC), and parietal TGC (P-TGC). Representing the main endocrine compartment of the placenta, the JZ produces a variety of hormones, growth factors, and cytokines that are essential for healthy progression of pregnancy (18, 19).

The decidua is modified endometrial tissue that undergoes pregnancy-induced changes for blastocyst implantation, spiral artery remodeling, and maternal immune tolerance at the maternal–fetal interface. In mice, the endometrial transformation of decidualization starts at embryonic day (E) 6.5 and is characterized by the proliferation of fibroblast cells, subsequent changes in the extracellular matrix (ECM), and increases in angiogenesis (20). Specific to mouse pregnancies and fully developing around E10.5, the MLAp is furthest away from the fetus and embedded within the myometrium of the uterine wall. The decidua and MLAp are the immune cell-rich tissues of the placenta, while few if any leukocytes are present in the fetal compartment (21). Seventy percent of all leukocytes within the combined decidua and MLAp are uterine natural killer (uNK) cells (also referred to as decidual NK or dNK cells) (20). These tissue-resident NK cells are noncytotoxic and the main cell type that drives remodeling of the high-capacitance maternal spiral arteries to lower-capacitance vessels with thinner arterial walls and wider lumens (22, 23). uNK cells are abundant in the mouse decidua early in pregnancy (E6.5), and peak in abundance between E10.5-E12.5 in both the decidua and MLAp (23, 24).

To identify mechanisms by which the gut microbiota affects placental and fetal development, we compared nonpregnant and pregnant GF, CONV-R, and CONV-D animals. Our goal was to define the effects of pregnancy on microbiota composition and gene expression in different regions of the small intestine and colon at time points that coincide with key stages of placental/fetal development, while concurrently examining how the microbiota influences placental development as a function of its different compartments and cell lineages. To do so, our analyses combined i) culture-independent characterization of gut microbiota composition, “bulk” (whole tissue segment) RNA sequencing (RNA-seq) of gene expression along the length of the intestine, and mass spectrometric assays of intestinal microbial metabolism (short chain fatty acid production), together with ii) placental bulk-RNA-seq, single-nucleus RNA-seq (snRNA-seq), histomorphometric, immunohistochemical, and proteomic assessments, and flow cytometry of decidual immune cell populations to assess overall placental development. Our results provide insights as to how structural and the expressed functional features of the fetal compartment of the placenta, and accompanying growth of the fetus, are modified by the gut microbiota.

## Results

### Intrauterine Growth Restriction in GF Compared to CONV-R Mice.

**Fig. 1.**
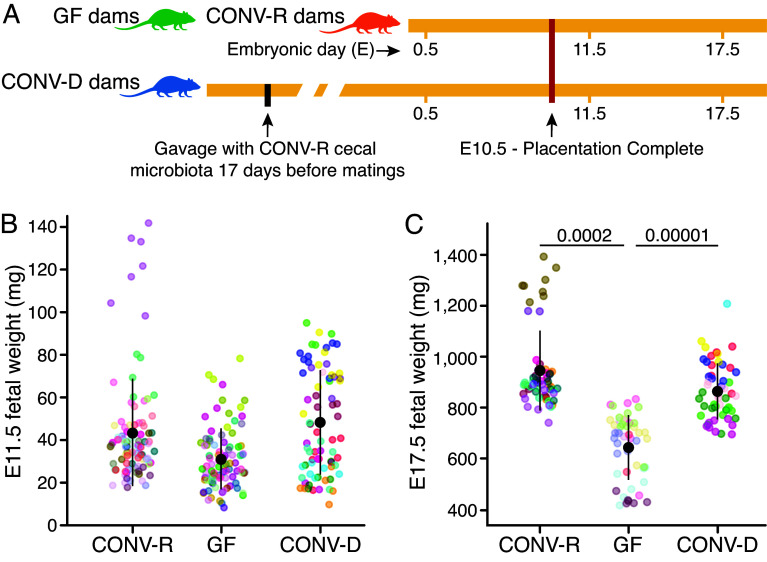
Intrauterine growth in CONV-R, GF, and CONV-D mice. (*A*) Experimental design. CONV-D mice were mated at least 17 d after gastric gavage with microbes. (*B*) Fetal weights were measured at E11.5 [CONV-R: 15 dams, 96 fetuses, 44.7 ± 25.0 mg (mean ± SD); GF: 15 dams, 91 fetuses, 32.0 ± 14.2 mg; CONV-D: 12 dams, 75 fetuses, 48.7 ± 24.0 mg] and (*C*) E17.5 (CONV-R: 8 dams, 58 fetuses, 948 ± 156 mg; GF: 8 dams, 49 fetuses, 644 ± 127 mg; CONV-D: 8 dams, 54 fetuses, 863 ± 110 mg). Linear mixed model for pairwise comparisons [*Fetal weight ~ Microbiota + Litter Size + (1 | Litter ID)*], with the BH correction for multiple comparisons. Each litter is represented by a different color of dots.

To assess the impact of the gut microbiota on fetal growth, we measured fetal weights in C57BL/6J GF and CONV-R mice at a time point when placentation is just completed (E11.5) (22), and at a time point near the end of gestation [E17.5; CONV-R C57BL/6J mice bear litters throughout E18.5-E20.5 (23, 25) (Fig. 1*A*). There were no statistically significant differences in fetal weights between the two groups at E11.5 (n = 83 to 96 fetuses representing 13 to 15 litters/group; *P* = 0.12; linear-mixed model with Benjamini–Hochberg (BH) correction for multiple pairwise comparisons) (Fig. 1*B*). However, a significant 32% reduction in fetal weight was evident in GF animals at E17.5 (mean value; n = 49 to 58 fetuses from 8 litters/group; *P* < 0.001) (Fig. 1*C*). These findings held for both male and female GF versus CONV-R fetuses (Dataset S1*A*). Moreover, there was no statistically significant difference in the sizes of litters between GF and CONV-R dams at either E11.5 (*P* = 0.93; Mann–Whitney–Wilcoxon rank test with BH correction) or E17.5 (*P* = 0.43) (Dataset S1*B*).

### Pregnancy and Gut Microbiota Composition.

To assess the effects of pregnancy on gut microbiota composition, we sequenced amplicons generated from variable region 4 (V4) of bacterial 16S rRNA genes present in cecal contents harvested from nonpregnant adult CONV-R females (n = 8) and comparably aged pregnant CONV-R dams at E11.5 (n = 6 animals/group) and E17.5 (n = 7 animals/group). All mice received a standard breeder chow diet ad libitum. A total of 334 amplicon sequence variants (ASVs) representing 7 phyla and at least 40 genera were identified (Dataset S2*A*). Alpha diversity measurements (Shannon and Simpson indices) were not significantly different as a function of pregnancy (Dataset S2*B*). Linear models of log-transformed absolute ASV abundances disclosed that, in comparison to cecal contents obtained from nonpregnant dams, there were no statistically significant differences at E11.5 and only a single ASV that exhibited a significant difference at E17.5 (reduction in ASV144, resolved to the genus *Muribaculum*, *P* = 0.019; Dataset S2*A*). Comparisons of ASV absolute abundances between E11.5 and E17.5 showed no significant differences for any of the taxa, supporting the compositional stability of the microbiota during pregnancy in these animals. Compositional stability was also evident when aggregating ASVs by family, order, class, or phylum (Dataset S2*C*).

### Intestinal Gene Expression Changes in Pregnancy Impacted by Colonization Status.

To examine the impact of pregnancy and colonization status on intestinal gene expression, we performed bulk RNA-sequencing (RNA-seq) of intact duodenal, jejunal, ileal, as well as colonic tissue segments harvested from nonpregnant GF and CONV-R mice and their pregnant counterparts at E11.5 and E17.5. Genes with statistically significant differences in their levels of expression (differentially expressed genes, DEGs) were identified using DESeq2 (26). Gene set enrichment analysis (GSEA) of Gene Ontology Biological Processes (GO-BPs) was performed with a focus on the leading edge differentially expressed transcripts (*Materials and Methods*). These methods were applied in two different ways. In Dataset S3, we tabulate the DEGs and GO-BP terms that result from comparisons across pregnancy (i.e., nonpregnant versus dams at E11.5 or E17.5) for each intestinal segment in CONV-R or GF dams. In Dataset S4, we list DEGs and GO-BP terms from direct comparisons of GF to CONV-R mice at each intestinal segment at each stage of pregnancy surveyed.

Gene sets positively enriched in the intestine with pregnancy (i.e., their leading edge transcripts show increased expression compared to nonpregnant mice) include those associated with GO-BP terms related to the vasculature [e.g., “Vascular Endothelial Growth Factor (VEGF) Signaling Pathway” and “Endothelial Cell Proliferation” with leading edge DEGs including *Kdr* (which encodes VEGFR2)], metal ion homeostasis [e.g., “Transition Metal Ion Transport” and “Inorganic Metal Ion Homeostasis” with leading edge DEGs that include *Slc31a1* (a copper transporter) and *Slc30a10* (a manganese transporter)], and small organic molecule metabolism [e.g., “Isoprenoid Metabolic Process” and “Steroid Metabolic Process” with leading edge DEGs including *Cyp3a44* (the cytochrome P450 3A4 enzyme)] (Dataset S3 *C* and *D*). Some differences were both region-specific and microbiota-dependent. For example, analysis of the duodenum disclosed statistically significant positive enrichment (GSEA) of gene sets related to i) BMP signaling at E11.5 (leading edge DEGs *Kdr* and *Ccn1*) and at E17.5 (leading edge DEGs *Kdr*, *Msx1*, and *Ccn1* with the latter two being microbiota-dependent) and ii) cell division (at E17.5 with leading edge DEGs *Cdc14a* and *Ereg*, both microbiota-dependent). The very minimal change in ASV composition documented in the gut microbiota during pregnancy precluded analysis of whether some or all of the observed temporal patterns of differential expression of these gene sets are related to specific community members.

Direct comparisons of pregnant GF versus CONV-R mice showed reduced enrichment (in GF animals) in GO-BP terms related to immune responses [e.g., “Antibacterial Humoral Response,” “Cytokine Production Involved in Immune Response,” and/or “Antigen Processing and Presentation of Peptide Antigen” with leading edge DEGs including genes encoding major histocompatibility class II components (e.g., *H2-Aa*), antimicrobial peptides (e.g., *Defa21*), antibody building blocks (e.g., *Jchain*), and/or pattern recognition receptors (e.g., *Nod2, Tlr7*)] across the intestine at one or both stages of pregnancy (Dataset S4 *E*–*H*). The GF versus CONV-R comparison yielded positive enrichment of GO-BP terms related to lipid metabolism [e.g., “Fatty Acid Metabolic Process,” “Steroid Metabolic Process,” and/or “Regulation of Cholesterol Metabolic Processes” with leading edge DEGs including genes encoding lipoproteins (e.g., *Apoa*), carboxylic ester hydrolases (e.g., *Ces1e*), and/or regulators of fatty acid metabolism (e.g., *Pparg*)] across all intestinal segments at one or both stages of pregnancy (Dataset S4 *E*–*H*).

We previously reported that GF mice studied at the conclusion of weaning (postnatal day 28) or in adulthood had a reduction in the density of the microvascular network underlying the small intestinal epithelium compared to their CONV-R counterparts, as judged by imaging with intravascularly injected FITC-dextran (27). Consistent with this finding, the GO-BP terms “Angiogenesis,” “Regulation of Vasculature Development,” “Endothelial Cell Proliferation,” and “Endothelial Cell Migration” were significantly enriched for leading edge transcripts with increased expression in the duodenum, ileum, and/or colon of CONV-R compared to GF dams at E11.5 and/or E17.5 (Dataset S4*I*). There were 63 DEGs in the leading edges of these gene sets, including four that were present at all three intestinal segments: *C3ar1*, which encodes a complement receptor demonstrated to be important for vascular endothelial growth factor receptor 2 (VEGFR2) signaling in endothelial cells (28); *Cybb*, which encodes an oxidase implicated in mediating VEGF-induced endothelial cell migration and proliferation (29); *Itgb2*, an integrin that mediates leukocyte-to-endothelial cell adhesion; and *Zc3h12a*, which encodes monocyte chemoattractant protein-induced protein 1 (MCPIP1 also known as Regnase-1), which has been linked to angiogenesis through increased HIF1a activity in endothelial cells (30).

We next collected all vasculature-related GO-BP terms associated with DEGs identified from the matrix of comparisons across pregnancy (Dataset S3*E*). In CONV-R dams, the biogeographic pattern of expression of these terms (e.g., “Endothelial Cell Proliferation” and “Angiogenesis”) varies as a function of position along the length of the gut and stage of pregnancy; positive enrichment is most prominent in the duodenum at both E11.5 and E17.5 (21 and 25 vascular-related GO-BP terms) but also manifests in the colon at E11.5 (4 terms) and in the jejunum and ileum at E17.5 (2 terms each). In GF mice positive enrichment of vasculature related GO-BP terms is limited to the duodenum at E11.5 (3 terms) and colon at E17.5 (2 terms) (Dataset S3*E*).

### Absence of the Gut Microbiota Reduces Fetal Endothelial Cell Markers in the Labyrinth Zone.

**Fig. 2.**
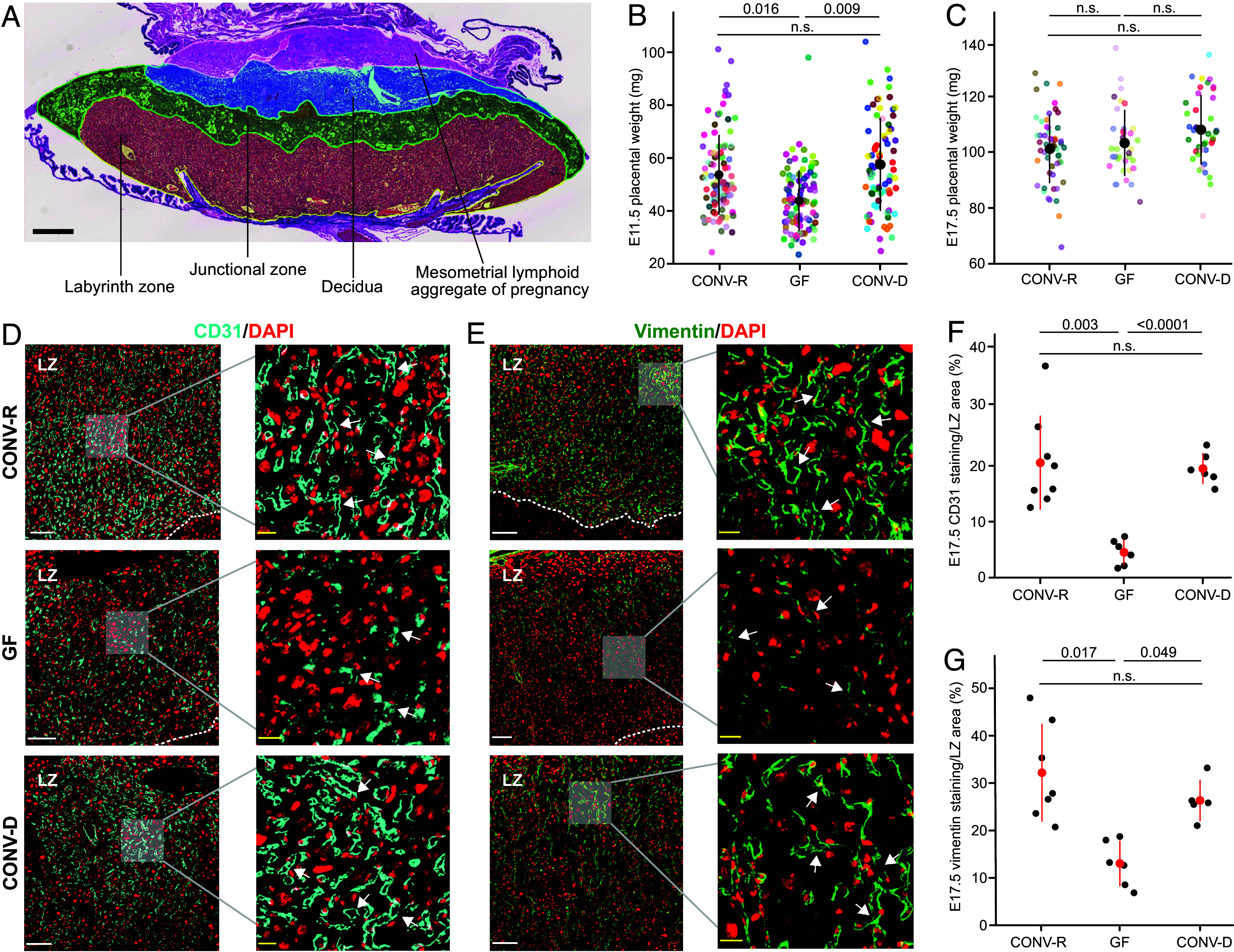
Effects of GF, CONV-R, and CONV-D microbiotas on placental weights and labyrinth zone vasculature. (*A*) The E17.5 mouse placenta is organized into the fetal labyrinth and junctional zones (LZ and JZ), and maternal decidua and mesometrial lymphoid aggregate of pregnancy (MLAp) (Scale bar, 500 µm). (*B* and *C*) Placental weights at (*B*) E11.5 (CONV-R: 15 dams, 95 placentas; GF: 13 dams, 92 placentas; CONV-D: 12 dams, 75 fetuses) and (*C*) E17.5 (CONV-R: 8 dams, 54 placentas; GF: 7 dams, 36 placentas; CONV-D: 8 dams, 41 placentas). Each litter is represented by different colored dots. Means and SD are shown. (*D* and *E*) Representative examples of immunostaining of (*D*) CD31 and (*E*) vimentin in the LZ of E17.5 placentas in CONV-R, GF, and CONV-D dams (*Left* scale bar, 100 µm; *Right* scale bar, 20 µm; white arrows indicate individual vessels). (*F* and *G*) The proportion of LZ area stained with (*F*) CD31 (n = 1 section/placenta; 1 to 2 placentas/dam; 7, 6, and 3 dams for CONV-R, GF, and CONV-D groups) and (*G*) vimentin across the different treatment groups (n = 1 section/placenta; 1 to 2 placentas/dam; 6, 4, and 3 dams for CONV-R, GF, and CONV-D groups). Adjusted *P*-values were defined using the linear mixed model [*% Staining ~ Microbiota + (1|Litter ID)*] for pairwise comparisons, with the BH correction applied. Means and SD are shown.

Placental weights were significantly reduced in GF compared to CONV-R mice immediately after placentation is complete at E11.5 (n = 84 to 95 fetuses representing 13 to 15 litters/group, *P* < 0.05, linear mixed model with BH correction). This difference was no longer significant by E17.5 (n = 36 to 54 fetuses representing 7 to 8 litters/group, *P* = 0.98) (Fig. 2 *B* and *C*). Histomorphometric measurements of spiral arteries in the decidua showed no significant difference in either the thickness of the arterial wall or the luminal area at E11.5 or E17.5 (n = 1 to 2 placentas/litter, 4 to 6 litters/treatment group) (*SI Appendix*, Fig. S1 *A–D*). Additionally, there was no statistically significant difference in the number of CD45 ^parenchymal+^ CD45^IV-^ CD3^−^ CD19^−^ NK1.1^+^ CD49a^+^ CD49b− uNK cells in the E11.5 decidual and MLAp compartments of GF and CONV-R mice (deciduas and MLAp were pooled from all placentas harvested from a given dam, n = 5 to 10 dams/treatment group, *P* = 0.95, Mann–Whitney–Wilcoxon test) (*SI Appendix*, Fig. S1 *E* and *F*). Thus, our subsequent analyses focused exclusively on the LZ, the major vasculature site in the fetal-derived mouse placenta.

Hematoxylin and eosin (H&E) staining of tissue sections demonstrated that in E17.5 placentas, there was no significant difference in the cross-sectional area of the LZ in GF versus CONV-R placentas (n = 8 sections/placenta, 1 to 2 placentas from each of 5 litters/group, *P* = 0.37, linear mixed model with BH correction) (*SI Appendix*, Fig. S2 *A* and *B*). To visualize the LZ vasculature more fully, we stained both E11.5 and E17.5 placental sections with antibodies against the endothelial cell marker CD31 (Fig. 2 *D* and *F* and *SI Appendix*, Fig. S2 *C*–*E*). At E11.5, there was no significant difference in positive CD31 staining in the LZ of GF compared to CONV-R placentas (n = 5 to 6 placentas from 5 litters/group, *P* = 0.63, linear mixed model with BH correction) (*SI Appendix*, Fig. S2 *C* and *E*). However, at E17.5, there was a significant decrease in positive CD31 LZ staining in GF compared to CONV-R placentas (n = 6 to 8 placentas from 3 to 6 litters/group, *P* < 0.01, linear mixed model with BH correction) (Fig. 2 *D* and *F* and *SI Appendix*, Fig. S2*D*). To confirm the E17.5 endothelial cell staining, we also stained for the E17.5 LZ fetal mesenchymal vascular marker vimentin. The results mirrored those obtained with CD31 and showed a significant decrease in positive E17.5 LZ vimentin staining in placentas from GF compared to CONV-R dams (n = 5 to 7 placentas from 3 to 5 litters/group, *P* < 0.05, linear mixed model with BH correction) (Fig. 2 *E* and *G* and *SI Appendix*, Fig. S2*F*). Given no significant difference in the LZ areas despite the difference in vascularization, we reasoned that another cell type might be increased in the GF LZ. This proved to be the case; immunostaining for the pan-trophoblast marker cytokeratin 7 revealed that at E17.5, trophoblast cells are more prominently represented in the LZ of GF than CONV-R animals (*SI Appendix*, Fig. S2*G*). Thus, when examined at late gestation, the lack of a gut microbiota is associated with reduced vascularization of the fetal-derived LZ and an increase in trophoblast cells.

### Aberrant Angiogenesis-Associated Signaling in GF Placentas.

Given the microbiota effects on placental vasculature, we next measured the levels of angiogenesis-associated proteins in the placentas of dams belonging to the different treatment groups. We dissected away the decidua from the fetal placenta at both E11.5 and E17.5 and performed all subsequent assays on the combined LZ and JZ areas. In E11.5 fetal placental homogenates, we observed significant *increases* in vascular endothelial growth factor A (VEGF-A), VEGF-C, follistatin, fibroblast growth factor 2 (FGF-2), stromal cell-derived factor 1 (SDF-1), and angiopoietin-2 in GF compared to CONV-R dams (n = 4 placentas/dam, 3 litters/group, *P* < 0.05, linear mixed model with BH correction) (*SI Appendix*, Fig. S3*A* and Dataset S5*A*). At E17.5, only angiopoietin-2, endoglin, and VEGF-A showed significant differences (*SI Appendix*, Fig. S3*B* and Dataset S5*A*). We next compared levels of these angiogenesis-associated proteins and placental and fetal weights. VEGF-A levels correlated negatively with placental weights in the CONV-R group but not in the GF group (Spearman R-coefficient −0.75 and 0.3 and *P* = 0.01 and 0.34, respectively, Dataset S5*B*). Furthermore, immunostaining of E11.5 and E17.5 placentas to ascertain the spatial localization of VEGF-A showed that it was primarily localized to the LZ in CONV-R mice in contrast to GF placentas where it was diffusely distributed throughout the LZ, JZ, and decidua (*SI Appendix*, Fig. S4 *A* and *B*). Angiopoietin-2 immunostaining was present mainly in the LZ regardless of colonization status at both stages of pregnancy (*SI Appendix*, Fig. S4 *C* and *D*).

**Fig. 3.**
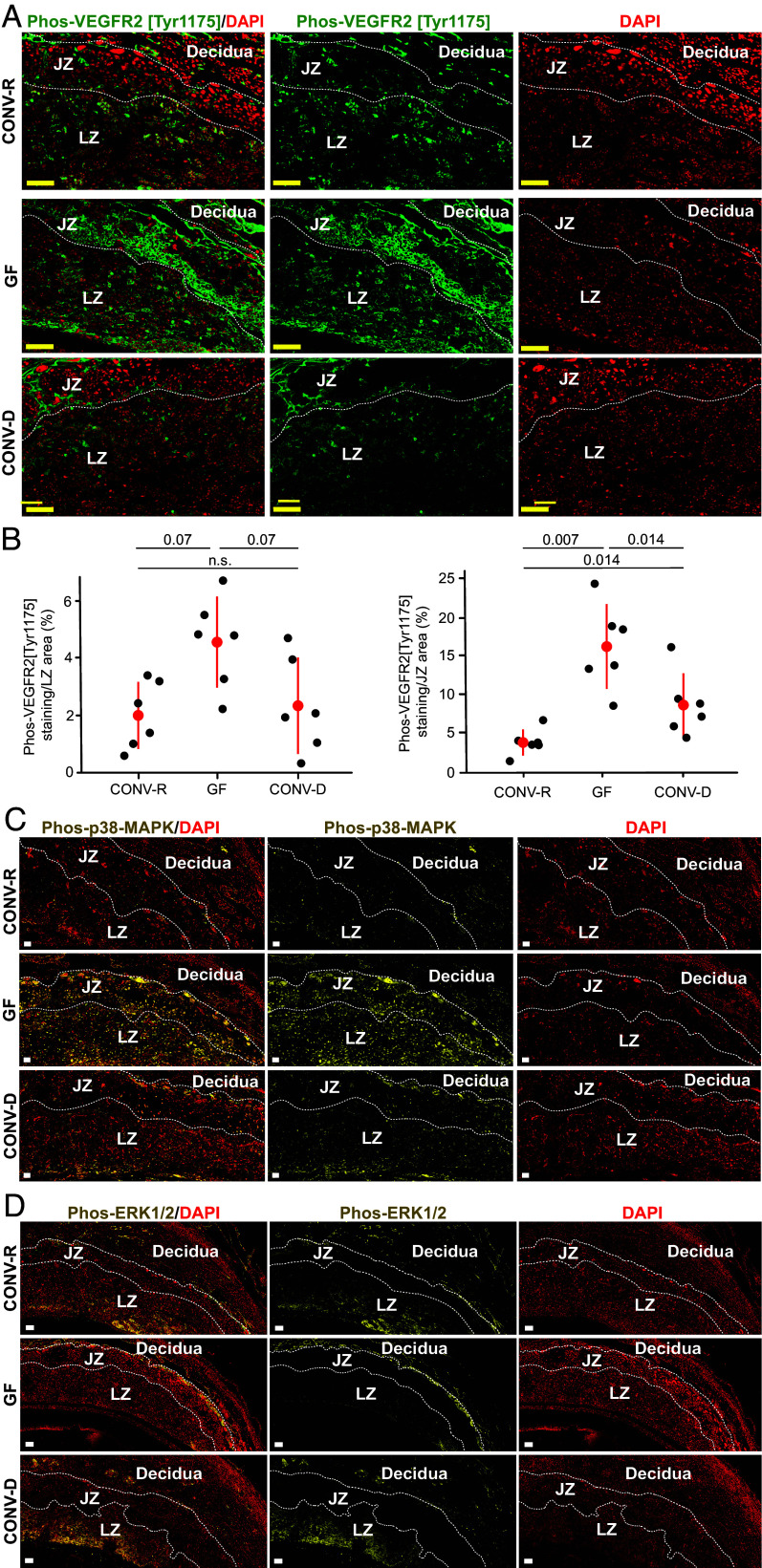
Immunohistochemical localization of phos-VEGFR2[Tyr1175], phos-p38-MAPK, and phos-ERK1/2 in placentas in E11.5 CONV-R, GF, and CONV-D mice. (*A*) Representative examples of immunostaining in E11.5 placentas for phos-VEGFR2[Tyr1175]. (Scale bar, 100 µm.) (*B*) Proportion of LZ and JZ area stained for phos-VEGFR2[Tyr1175] across different treatment groups (n = 1 section/placenta, 1 to 2 placentas/litter, 5 to 6 litters/treatment group). Adjusted *P*-values were defined using the linear mixed model [*% Staining ~ Microbiota + (1 | Litter ID)*] for pairwise comparisons, with the BH correction applied. Means and SD are shown. (*C* and *D*) E11.5 placentas immunostained for (*C*) phos-p38-MAPK (Scale bar, 100 µm) and (*D*) phos-ERK1/2 (Scale bar, 100 µm). Images shown in Panels *C* and *D* are representative of those found in 3 to 4 sections/placenta,1 placenta/litter and 3 litters/treatment group.

The presence of increased levels of angiogenic proteins in the fetal compartment of GF placentas yet deficient LZ vascularization at E17.5 suggests a breakdown in angiogenic signaling pathways. To explore this possibility, we focused on the VEGF-A pathway because VEGF-A was significantly increased in GF compared to CONV-R placentas at E11.5 and E17.5 and showed correlations with placental weights in the CONV-R group. Within the placenta, the majority of VEGF-A signaling in vascular endothelial cells is through VEGFR2 (31). Phosphorylation of VEGFR2 at tyrosine 1175 (phos-VEGFR2[Y1175]) activates multiple kinase cascades including the MAPK (mitogen-activated protein kinase) and ERK (extracellular signal-regulated kinase) pathways that then promote endothelial cell migration and proliferation (32, 33). Quantification of phos-VEGFR2[Y1175] by immunostaining showed that GF placentas have increased LZ and JZ staining compared to CONV-R placentas (n = 1 section/placenta, 1 to 2 placentas/litter, 5 to 6 litters/treatment group, *P* = 0.07 and 0.007 respectively, linear mixed model with BH correction) (Fig. 3 *A* and *B*). Immunostaining demonstrated that phos-p38-MAPK was also increased in the LZ of E11.5 GF compared to CONV-R placentas (n = 3 to 4 sections/placenta, 1 placenta/litter, 3 litters/treatment group) (Fig. 3*C*). However, immunostaining revealed marked reduction in phos-ERK1/2 in the LZ of E11.5 GF versus CONV-R placentas (n = 3 to 4 sections/placenta,1 placenta/litter, 3 litters/treatment group) (Fig. 3*D*). These results suggest that VEGF-A/VEGFR2 signal transduction in GF placentas is insufficiently transmitted to the ERK1/2 phosphorylation step associated with endothelial cell proliferation (34, 35), at least partially explaining the observed deficient placental vascularization despite a seemingly compensatory upregulation in levels of angiogenic ligands.

Low levels of maternal serum progesterone correlate with IUGR in human pregnancy (36) as well as in mouse and rat models (37, 38). We detected a significant increase in progesterone at E11.5 in placental homogenates prepared from GF compared to CONV-R dams even though placental and fetal weights were reduced (n = 4 placentas/dam, representing 3 litters/group, *P* < 0.001, linear mixed model with BH correction). In contrast, there was no significant difference in progesterone levels between GF and CONV-R placentas at E17.5 (n = 4 placentas/dam, representing 3 litters/group, *P* = 0.33) (*SI Appendix*, Fig. S5 *A* and *B*). Immunostaining of the progesterone receptor in E11.5 placentas showed expression in the decidua, with similar staining in GF and CONV-R animals (*SI Appendix*, Fig. S5 *E* and *F*). Given the lack of progesterone receptor expression in the LZ and JZ, the difference in progesterone levels is unlikely to directly underlie the differences in fetal placental vascularization between treatment groups. Moreover, given that progesterone has been linked to upregulated VEGF-A expression and placenta vascularization (39), the elevated levels of this hormone observed in the placentas of GF E11.5 mice may be part of a compensatory, albeit ineffective, response to disrupted VEGF-A/VEGFR2 signal transduction in these animals.

Estradiol, another hormone essential for the initiation and continuation of gestation, also promotes angiogenesis. However, placental levels of estradiol were not statistically different between GF and CONV-R dams at either E11.5 or E17.5 (n = 4 placentas/dam, representing 3 litters/group, *P* = 0.27 and *P* = 0.85, respectively) (*SI Appendix*, Fig. S5 *C* and *D*).

### Effects of Colonization Status and Gestational Age on Intestinal Short Chain Fatty Acid Levels.

Short chain fatty acids are produced by bacterial fermentation in the intestine. A prior study reported that administration of a combination of acetate, propionate, and butyrate to CONV-R dams that had received antibiotics or a protein deficient diet throughout pregnancy, improved their impairments in placental vascularization (15). We used gas-chromatography-mass spectrometry of cecal contents to further characterize how microbial production of SCFAs is related to developmental changes in placental vascularization. The results revealed no statistically significant differences in levels of acetate, propionate, or butyrate between nonpregnant CONV-R dams and those belonging to the E11.5 and E17.5 groups. As expected, analysis of cecal contents from GF mice showed low to undetectable levels of SCFAs at both stages of pregnancy (Dataset S6). These functional results align with the compositional stability of the microbiota during pregnancy in CONV-R mice.

### Colonizing GF Dams with Microbiota from CONV-R Dams Rescues Placental Weight and Angiogenesis.

We next transplanted cecal contents pooled from three nonpregnant CONV-R mice to nonpregnant GF animals (Fig. 1*A*). Before starting timed matings, we allowed a minimum of 17 d for colonization of their intestines and then assessed the conventionalized (CONV-D) mice at E11.5 (n = 69 fetuses, representing 11 dams) and at E17.5 (n = 42 fetuses, representing 6 dams). Eighty-seven percent of the 334 ASVs detected in cecal microbiota of CONV-R mice were detected in the cecal microbiota of CONV-D dams (Dataset S1*A*). Predictably, colonization of GF mice greatly augmented SCFA production (Dataset S6).

Interestingly, CONV-D dams phenocopied CONV-R dams with respect to i) fetal weights at E17.5 (Fig. 1*C*), ii) placental weights at E11.5 (Fig. 2*B*), iii) LZ CD31, vimentin, and cytokeratin-7 immunostaining at E17.5 (Fig. 2 *D*–*G* and *SI Appendix*, Fig. S2 *D, F,* and *G*), iv) placental levels of VEGF-A, VEGF-C, FGF-2, and SDF-1 at E11.5 (*SI Appendix*, Fig. S3*A*) and endoglin and VEGF-A at E17.5 (*SI Appendix*, Fig. S3*B*), v) progesterone at E11.5 (*SI Appendix*, Fig. S5*A*), and vi) the fetal placental pattern of VEGF-A, phos-VEGFR2[Y1175], phos-p38-MAPK, and phos-ERK1/2 staining at E11.5 (Fig. 3 *A*–*D*). Thus, introducing cecal communities from CONV-R donors into GF mice with the same genetic background “rescued” several facets of placental development, providing direct evidence of the effects of the gut microbiota on placental and fetal development.

### GF Placentas Have Decreased Expression of Junctional Zone-Specific *Psg* and *Ceacam* Glycoprotein Genes.

To assess the effects of colonization on other facets of placental biology, we performed bulk RNA-seq of placentas obtained from GF, CONV-R, and CONV-D mice at E11.5 (n = 4 fetuses/dam, 3 dams/group). As with the placental homogenates used for quantification of angiogenic proteins, we removed the decidual tissue from the LZ and JZ to focus on contributions from the fetal-derived placenta. Differential gene expression was defined using DESeq2 and GSEA was employed to identify Hallmark and Gene Ontology (GO) terms enriched in DEGs. The results of this matrix of comparisons are presented in Dataset S7. Compared to CONV-R and CONV-D placentas, GF placentas had significantly reduced expression of genes encoding many glycoproteins belonging to the pregnancy-specific glycoprotein (PSG) and carcinoembryonic antigen-related cell adhesion molecule (CEACAM) families (40, 41) (*SI Appendix*, Fig. S6 and Dataset S7*A*). PSGs are produced by fetal-derived placental trophoblasts; the few studies that have explored the role of PSGs in pregnancy support their function in autocrine and paracrine maternal immunomodulatory, angiogenic, and cell adhesion functions (40). *Psg16*, *Psg21*, and *Psg23*, which have significantly reduced expression in GF compared to both CONV-R and CONV-D placentas, are produced by JZ-specific spongiotrophoblast (SpT) cells (40). In mice, PSG17 and PSG22 bind glycosaminoglycans (GAGs), specifically heparin and heparan sulfate proteoglycans, indicative of their proangiogenic function (42, 43). An in vitro study showed that PSG23 induces transforming growth factor beta 1 (TGFB1) and VEGF in mouse endothelial cells derived from the yolk sac and in cells with characteristics of LZ trophoblasts—both essential cell types for establishing fetal blood supply during placentation (44).

### Single Nucleus-RNA-Seq Demonstrates Endothelial and Stromal Cell Perturbations in GF Placentas.

**Fig. 4.**
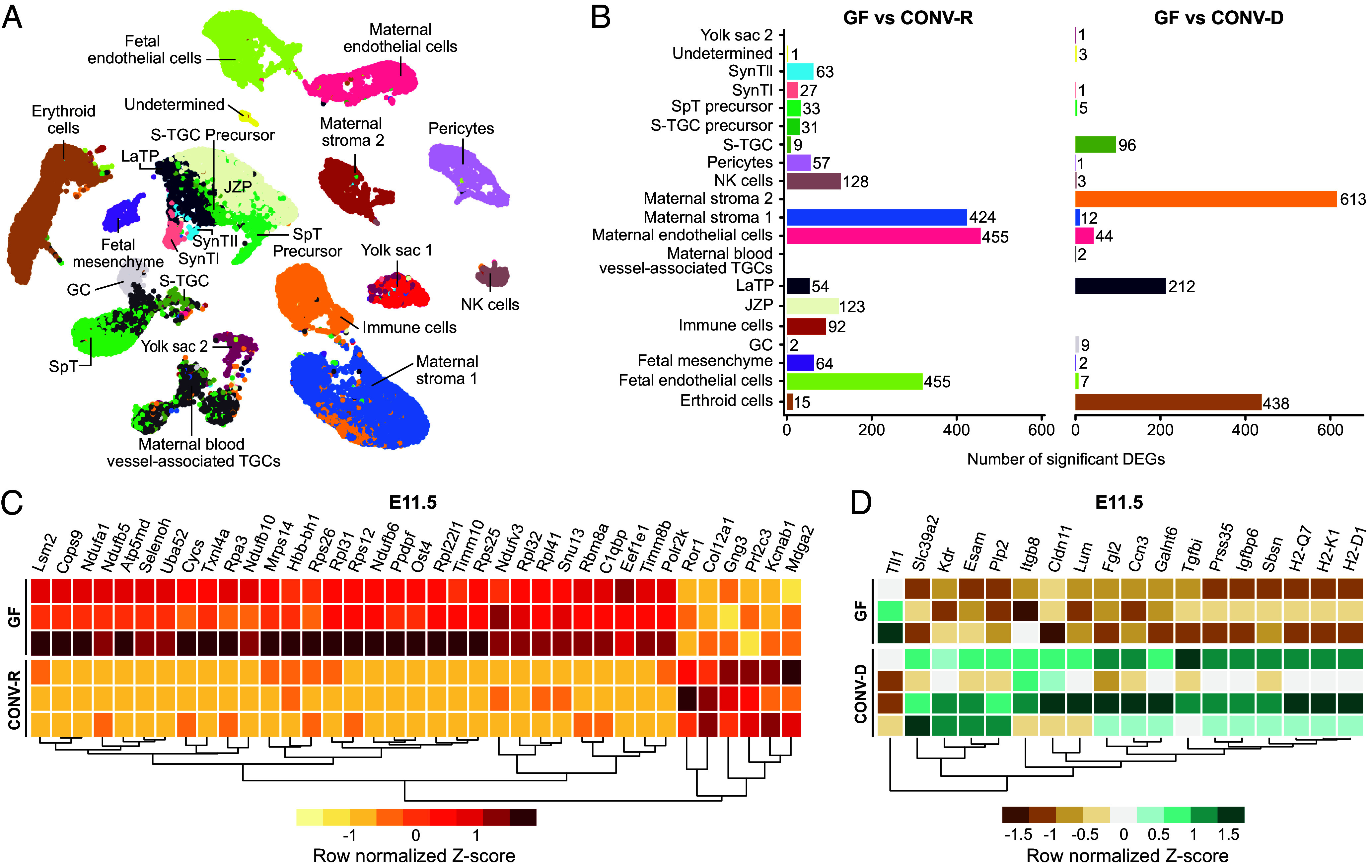
snRNA-seq of E11.5 CONV-R, GF, and CONV-D placentas. (*A*) UMAP representation of 32,571 nuclei across ten E11.5 placentas (3 CONV-R, 3 GF, and 4 CONV-D) clustered and assigned to 21 cell types. (*B*) Number of DEGs identified in each cell type by pseudobulk analysis when comparing GF to CONV-R or CONV-D placentas (DESeq2, |log_2_(fold-difference)| > 0.5, adjusted *P*-value < 0.05, Wald test with BH correction). (*C*) DEGs identified when comparing GF to CONV-R maternal endothelial cells (log_2_(fold-difference) < −1.5 or > 5.5). (*D*) DEGs identified when comparing GF to CONV-D maternal stroma 2 cells (|log_2_(fold-difference)| > 2).

To achieve a more detailed understanding of the placental cell lineages responsible for microbiota-dependent responses, we performed snRNA-seq of placentas harvested from GF, CONV-R, and CONV-D dams at E11.5 and E17.5. We reasoned that a snRNA-seq rather than a single cell RNA-seq approach would avoid the difficulties with capturing the large multinucleated syncytiotrophoblasts of the LZ, which are often underrepresented in single cell RNA-seq datasets (45). snRNA-seq was conducted using placentas in which the decidual tissue was removed. For the E11.5 snRNA-seq dataset, we obtained 32,571 nuclei with a median of 2,352 expressed genes detected per nucleus after quality control (*Materials and Methods*). For the E17.5 dataset, we obtained 36,918 nuclei with a median of 2,199 expressed genes detected per nucleus. See Fig. 4*A* and *SI Appendix*, Fig. S9*A* for cell clusters identified, and *SI Appendix*, Figs. S7 and S8 plus Datasets S8*A* and S9*A* for genes used to identify/define each cell cluster. Maternal compartment cells included two subtypes of stromal cells, endothelial cells plus immune cells, with E11.5 placentas including a NK cell cluster. The epithelial (E17.5) and pericyte (E11.5 and E17.5) cell clusters represent a mixture of both fetal and maternal-origin cells. Fetal compartment cell types include i) trophoblast precursor cells at E11.5 [labyrinth trophoblast progenitors (LaTP), junctional zone precursors (JZP), SpT precursors plus sinusoidal trophoblast giant cells (S-TGC) precursors]; ii) fully differentiated trophoblast cell types at both E11.5 and E17.5 such as SpTs, glycogen cells (GCs), S-TGCs, maternal blood vessel-associated TGCs (MBV-TGCs), and syncytiotrophoblast types I and II (SynTI, SynTII)]; iii) yolk sac cells; iv) erythroid cells; v) fetal mesenchymal cells; and vi) fetal endothelial cells. Due to similarities in gene expression in spiral artery-associated (SpA), canal (C), and channel (Ch) trophoblast giant cells, those clusters at E11.5 and E17.5 were considered to represent a mixture of TGCs and designated MBV-TGCs. Both E11.5 and E17.5 had two undetermined clusters, with the E17.5 cluster being a trophoblast cell type based on expression of the pan-trophoblast marker *Krt18* (46), SpT markers such as *Tfrc* and *Fabp3,* and SynTII markers including *Vegfa* and *Synb,* but with high expression of *Cd9* and *Cd63* which encode extracellular-vesicle (EV) exosome tetraspanins (47). These undetermined trophoblasts, or EV-associated trophoblasts, have been shown in human placentas to release angiogenesis-related proteins including VEGF-A, FGF-2, and PDGF (48). Moreover, high exposure to plasma exosomes derived from feto-maternal uterine tissue late in gestation is associated with preterm birth in mice (49).

Using the approach described in *Materials and Methods*, we identified genes with significant differences in their expression in GF compared to CONV-R or CONV-D placental cell lineages (see Datasets S8*B* and S9*B* for lists of all DEGs in each cell cluster as a function of colonization state and gestational age). E11.5 cell clusters demonstrated more DEGs across all clusters compared to E17.5 cell clusters (Fig. 4*B* and *SI Appendix*, Fig. S9*B* and *E*). The GF versus CONV-D and the GF versus CONV-R comparisons had a divergent composition of DEGs between cell clusters at both time points. At E11.5, the greatest numbers of DEGs in the GF versus CONV-R comparison were in the maternal endothelial, maternal stroma 1, and fetal endothelial cell clusters, whereas for the GF versus CONV-D comparison, the top three clusters ranked by their number of DEGs were maternal stroma 2, erythroid, and LaTP cells (Fig. 4*B*).

### E11.5 GF Versus CONV-R Placentas.

Comparing CONV-R and GF placentas at E11.5, maternal endothelial cells demonstrated the greatest number of DEGs. Focusing on the largest fold differences (log_2_(fold-difference) > 5.5) yielded 31 genes involved in several fundamental processes that govern cellular homeostasis (Fig. 4*C*). They include genes whose products are present in the mitochondrial respiratory chain complex (*Ndufa1, Ndufb5, Ndufb6, Ndufb10*), involved in mitochondrial ATP synthesis/transport (*Atp5mk, Timm10, Ndufv3*), and mitochondrial protein translation (*Mrps14, C1qbp*), as well those that regulate redox state and reactive oxygen species (ROS) (*Selenoh, Cycs, Eef1ef*). They also included genes involved in cell cycle regulation and differentiation (*Selenoh, Cycs, Rpa3, Ppdpf, Eef1ef*), spliceosome formation (*Lsm2, Txnl4a, Rps26, Snu13, Rbm8a*), transcription (*Hbb-bh1, Polr2k*), protein translation [*Rps26, Rpl31, Rps12, Rpl22l1, Rpl32, Rpl41, Eef1* plus other structural components of ribosomes (*Uba52, Mrps14, Rps26, Rpl31, Rps12, Rpl22l1, Rps25, Rpl32, Rpl41*)], as well as posttranslational protein modifications (*Cops9, Ost4*) (Fig. 4*C* and Dataset S8*C*). These themes are highlighted when performing GSEA using the GO “Cellular Component,” “Biological Process,” and “Molecular Function” annotated databases (Dataset S8*D*). GSEA of bulk RNA-seq data generated from E11.5 placentas using the same databases shows a similar pattern of ribosomal and translation related terms being enriched in GF compared to CONV-R and CONV-D placentas (Dataset S7*B*).

Like the maternal endothelial cells’ DEGs, fetal endothelial cells had upregulated DEGs (|log_2_(fold-difference)| > 5.5) associated with mitochondrial respiration (*Ndufb6*), mitochondrial cytochrome P450 protein reactions (*Fdx1*), mitochondrial gene transcription (*Mrps34*), and mitochondrial gene translation (*Prl7b1*, *Alyref*) (Dataset S8*E*). GSEA of leading-edge transcripts revealed a similar profile of GO terms to *maternal* endothelial cells including increased of genes classified under the terms “Cytoplasmic Ribosomal Structure/Function,” “Translation,” and “Peptide Biosynthetic/Metabolic Processes,” but with reduced expression of genes associated with unique terms including “Carbohydrate Derivative Metabolic Process,” “Mitochondrial Respiration and Respiratory Chain Assembly,” “Small Molecule Metabolic Process,” and “Energy Derivation by Oxidation of Organic Compounds” (Dataset S8*F*).

In addition to fetal and maternal endothelial cells, GF fetal mesenchymal and maternal stroma 1 cells had DEGs (compared to the corresponding CONV-R cell clusters) that are related to promoting angiogenesis (Dataset S8*B*). The GF fetal mesenchymal cluster displayed increased expression of *Id2* (inhibitor of DNA binding 2) and *Mif* (macrophage migration inhibitory factor). ID2 promotes tumor angiogenesis by functioning as a master regulator of VEGF (50) which activates endothelial cells to proliferate and differentiate (51). While primarily associated with its role as a proinflammatory cytokine, MIF has potent proangiogenic properties; in vitro it stimulates endothelial cell migration, proliferation, and tube formation and acts as a potent inducer of angiogenic factors in tumors, such as VEGF, CXCL5, and CXCL8 (52).

The GF maternal stroma 1 cell cluster exhibited increased expression of *Uqcrb* (ubiquinol-cytochrome C reductase binding protein) and *Pfn1* (profilin 1). UQCRB, of mitochondrial complex III, induces angiogenesis in vitro using a protein transduction domain conjugated to a UQCRB fusion protein by stabilizing hypoxia inducible factor 1α (HIF-1α) and increasing downstream VEGF expression (53). PFN1, an actin-binding protein, is involved in endothelial migration and proliferation. Silencing of PFN1 leads to reduced endothelial cell adhesion that inhibits capillary morphogenesis and ECM invasion (54, 55). The involvement of the mesenchymal and stromal compartments highlights the importance of nonendothelial cell clusters on propagating microbiota-directed effects on placental angiogenesis.

### E11.5 GF Versus CONV-D Placentas.

In the GF versus CONV-D comparison, maternal stroma 2 cells had the most DEGs; the top 18 with the greatest changes (|log2(fold-difference)| > 2) were genes associated with ECM maintenance (*Lum*, *Fgl2*, *Ccn3*, *Galnt6*,*Tgfbi*, *Prss35*, *Igfbp6*), cell adhesion (*Esam*, *Cldn11*), MHC class I complex members (*H2-Q7*, *H2-K1*, *H2-D1*), and vascular development (*Kdr*, *Sbsn*) (Fig. 4*D* and Dataset S8*G*). Similarly, GSEA revealed a decrease in GO terms related to “Extracellular Space,” “Cell Adhesion’, “Cell Surface,” “Positive Regulation of Signal Transduction,” and “Regulation of Response to Stress” (Dataset S8*H*). The maternal stroma 2 cluster represents a novel senescent decidual cell cluster (56). Compared to maternal stroma 1 cells, this cell cluster is characterized, both by Stadtmauer et al. (56) and our dataset, as having low levels of expression of genes encoding products with endocrine functions [*Pgr* (progesterone receptor) and *Esr1* (estrogen receptor 1)] and high levels of expression of oxidative stress-related genes (*Gpx3*, *Cryab*, and *Ctsk*) relative to the maternal stroma 1 cells. Maternal stroma 2 is analogous to progesterone-resistant decidual cells identified in humans (56, 57). The reduction in expression of *Kdr*, which encodes VEGFR2, in GF compared to CONV-R placental stroma 2 cluster aligns with our protein quantification and immunohistochemical findings that found evidence of aberrant VEGF-A signaling in GF placentas (Fig. 4*D*).

### E17.5 GF Versus CONV-R and CONV-D Placentas.

In contrast to the E11.5 snRNA-seq data, there were markedly fewer DEGs at E17.5. Across all cell types at E11.5, the GF versus CONV-R comparison yielded 1,897 DEGs and the GF versus CONV-D comparison 1,449 DEGs, while at E17.5 the GF versus CONV-R comparison yielded 125 DEGs and the GF versus CONV-D comparison 21 DEGs (Fig. 4*B* and *SI Appendix*, Fig. S9*B*). Thus, our analysis of the E17.5 snRNA-seq results focused primarily upon the differences between GF and CONV-R placentas. Unlike at E11.5, we were unable to obtain the transcriptional resolution to separate E17.5 maternal and fetal endothelial cell clusters (Fig. 4*A* and *SI Appendix*, Fig. S9*A*). However, similar to the E11.5 maternal and fetal endothelial cell clusters, at E17.5 GF endothelial cells have nine DEGs associated with ribosomal function (*Rpl35*, *Rpl32*, *Rpl26*, *Rps7*, *Rps13*, *Rpl27*, *Rps26*, *Rpl12*, *Rpl36*) and five related to mitochondrial functions (*Uqcc2*, *Atp5mpl*, *Atp5md*, *Prdx1*, *Cox8a*) with greater expression in CONV-R endothelial cells (*SI Appendix*, Fig. S9*C* and Dataset S9*C*; |log_2_(fold-difference)| > 0.5). Most DEGs in the E17.5 GF versus CONV-R comparison were in the maternal stroma 1 cluster; the top 19 DEGs in this cluster (by log_2_(fold-difference)) encompassed a large range functions including placental development (*Maged2*, *Rtl1*) and regulation of angiogenesis (*Edn1*, *Arhgef26*, *Ptprb*, *Rtl1*, *Lama1*) (*SI Appendix*, Fig. S9*D* and Dataset S9*D*; |log_2_(fold-difference)| > 0.5). Together, these findings suggest that at this late stage of pregnancy, the microbiota has direct effects not only on the placental vasculature, but also on maternal stroma cells that communicate with endothelial cells to control angiogenesis.

### E11.5 and E17.5 CONV-D Versus CONV-R Placentas.

The E11.5 CONV-D versus CONV-R placental snRNA-seq dataset contained a total of 1,456 DEGs with |log_2_(fold-difference)| > 0.5) (*SI Appendix*, Fig. S9*E* and Dataset S8B). Similar to the E11.5 GF versus CONV-R and CONV-D comparisons, the largest number of DEGs was in the maternal and fetal endothelial cell and maternal stromal 1 and 2 cell clusters (*SI Appendix*, Fig. S9*E*). As in the GF versus CONV-D comparison, there was also a substantial number of DEGs in the trophoblast progenitor cluster LaTPs (n = 278) (Fig. 4*B* and *SI Appendix*, Fig. S9*E*). GSEA highlighted similar ribosomal-related pathways as in the E11.5 GF versus CONV-R and CONV-D comparisons for maternal and fetal endothelial cells; i.e. the CONV-D maternal endothelial cell cluster had a decrease in GO terms related to “Small Ribosomal Subunit’, “Ribosomal Subunit,” “Organonitrogen Compound Biosynthetic Process,” “Ribosome,” and “Structural Constituent of Ribosome” while the CONV-D fetal endothelial cluster had a decrease in GO terms related to “Ribosome,” “Cytoplasmic Translation,” “Ribosomal Subunit,” “Large Ribosomal Subunit,” and “Peptide Biosynthetic Process” for the fetal endothelial cell cluster compared to the CONV-R clusters (Dataset S8 *I* and *J*). At E17.5, there are far fewer DEGs in the CONV-D versus CONV-R comparison (n = 33 across all clusters with the greatest number in the endothelial cell cluster) (*SI Appendix*, Fig. S9*B*). In the CONV-D condition, there was greater expression of genes associated with both ribosomal (*Eef1e1*, *Rps13*, *Rpl35*) and mitochondrial (*mt-Atp6*, *Uqcc2*) functions (Dataset S9*B*, |log_2_(fold-difference)| > 0.5). These results from the E11.5 and E17.5 comparisons indicate that the placenta in a CONV-D mouse occupies an intermediate state between the GF and CONV-R physiological conditions.

## Discussion

IUGR is a global health challenge that requires additional insights into its pathogenesis as well as new therapeutic targets. We have applied histological, immunohistochemical, proteomic, and RNA sequencing approaches to GF, CONV-R, and CONV-D mice to characterize the effects of pregnancy on gut microbiota composition and gene expression in different regions of the intestine at time points that coincide with key stages of placental/fetal development, while simultaneously examining how the microbiota influences the different compartments and cell lineages of the placenta. Our findings illustrate the responsiveness of the biologic state of placental cell lineages at various times during gestation to the gut microbiota, with pronounced effects on angiogenic pathways, particularly in the LZ of the fetal compartment, which directly participates in maternal–fetal exchange of nutrients and waste.

Our findings, obtained at the time point when placentation has just been completed (E11.5) and at a late stage of pregnancy (E17.5), demonstrate a fetal growth defect in GF mice that develops during this period; this finding is consistent with a prior study that showed a significant difference at E16.5 (58). The GF state is accompanied by decreases in GF placental weight at E11.5 that is associated with an elevation of VEGF-A, elevated phosphorylation of the VEGF-A target receptor VEGFR2, elevation of downstream p38-MAPK phosphorylation, yet a lack of downstream ERK1/2 phosphorylation. Abnormal elevation of p38-MAPK phosphorylation has been associated with antiangiogenic phenotypes including endothelial cell cycle arrest (59). Additionally, ERK1/2 phosphorylation is associated with endothelial cell proliferation (34, 35, 60).

Our snRNA-seq analyses demonstrate that in E11.5 GF mice, maternal and fetal endothelial cells exhibit signatures of cellular stress, with broad upregulation of mitochondrial and ribosomal genes. Our findings of perturbed VEGFR2 signal transduction hint at a potential explanation for the increase in mitochondrial and ribosomal genes. Previous studies have shown that VEGFR2 blockade via the VEGFR2 inhibitor Ki8751 in both breast cancer and glioblastoma cancer cell lines leads to increases in mitochondrial proteins, with increased mitochondria mass, cellular oxygen production, and reactive oxygen species in a glioblastoma line (61, 62). Guo et al. (2024) also found that mitochondrial biogenesis and increased oxidative phosphorylation respiration via VEGFR2 inhibition led to suppressed cell proliferation and apoptosis (61). Future studies are needed to further elucidate how the presence of a microbiota influences VEGFR2 signal transduction and endothelial mitochondrial function.

Our results highlight another previously unexplored role of the gut microbiota; namely, its ability to impact each placental compartment (the fetal-derived LZ and JZ and the maternal-derived decidua) differently. Although uNK cells play a central role within the maternal decidua in remodeling vascular supply to the fetus (20, 63), we did not find significant changes in the number of decidual uNK cells at E11.5 or in decidual vascular morphometrics in GF dams at E11.5 or E17.5. Therefore, our analysis of the microbial effects on placental development focused on the fetal placental compartment. Our bulk RNA-seq results demonstrate that in addition to the perturbations in angiogenesis in the LZ of GF placentas, there is decreased expression of a number of JZ-specific *Psg* and *Ceacam* genes in the GF compared to colonized states. These genes encode glycoproteins belonging to the CEA family (41). Of note, in humans, transcriptomic studies of term placentas have demonstrated decreased expression of *Ceacam6* in cases of IUGR (64). In addition, levels of PSGs in the maternal circulation correlate with intrauterine growth (65); e.g., serum levels of PSG1 are decreased in the first trimester in pregnancies associated with small for gestational age outcomes, spontaneous preterm delivery, and pre-eclampsia (66, 67). PSG and CEACAM are postulated to have immunomodulatory and proangiogenic functions (40, 41), but their exact roles in the placenta remain unclear. The mechanism by which the microbiota affects expression of *Psg* and *Ceacam* in the placental JZ is unclear and requires further investigation.

The importance of the gut microbiota in supporting intestinal vasculature was previously demonstrated in P28 and adult mice (27). Others identified microbiota-directed glycosylation of tissue factor (TF), the membrane receptor that activates the extrinsic coagulation pathway, as inducing a cascade of molecular changes in small intestinal villus enterocytes that increase TF’s procoagulation activity and phosphorylation of its cytoplasmic tail, which in turn increases vessel density in the intestines (68). Anti-TF treatment in CONV-D mice also reduced expression of *Pecam1* (CD31) and *Ang1* (angiopoietin-1). We now extend this relationship between the microbiota and vasculogenesis in the small intestine and colon to pregnancy, where gene sets related to blood vessel formation were significantly upregulated in pregnant CONV-R compared to GF mice but less so in their nonpregnant counterparts. Increase in intestinal blood vessel formation in pregnancy could serve to support the intestinal villus lengthening known to occur during this period (69–72). Our observation that there was a minimal statistically significant change in the absolute abundance of only one bacterial ASV and no significant changes in intestinal levels of short chain fatty acids during pregnancy in CONV-R dams emphasize the need to look beyond “who’s there” in the gut microbiota and perform analyses of their expressed functions. The latter could include assessments of (i) gene expression ( e.g., to identify metabolic pathways that may be differentially expressed as a function of pregnancy), ii) metabolic output (informed in a hypothesis-based manner by the results of the metatranscriptomic analysis), and iii) immune function (to better understand the cross-talk between signals emanating from the microbiota and uNK cells, in addition to other decidual immune cell populations). These types of studies may be particularly informative if gut microbial communities from healthy mothers and undernourished mothers with a history of IUGR are introduced into GF female mice prior to and during pregnancy. In summary, our results provide a rationale for using gnotobiotic models to further elucidate underlying mechanisms, identify candidate therapeutic targets, and develop candidate microbiota-directed therapeutics for IUGR.

## Materials and Methods

### Mouse Experiments.

All experiments involving mice were performed using protocols approved by the Washington University Animal Studies Committee. Germ free (GF), conventionalized (CONV-D), and conventionally raised (CONV-R) specific pathogen-free C57BL/6J mice were housed in plastic flexible film gnotobiotic isolators (Class Biologically Clean Ltd., Madison, WI) at 23 °C under a strict 12-h light cycle (lights on at 0700 h), with autoclaved paper “shepherd shacks” kept in each cage to facilitate nesting and enrichment. Autoclaved standard chow (Lab Diet 5021) and sterile water were provided ad libitum. 8 to 19-wk-old primiparous female mice were subjected to timed matings in which 1 to 2 females were placed in a cage with 1 to 2 males. After 12 to 18 h [embryonic day (E)0.5], females were checked for the presence of a vaginal plug, separated from the males and placed back in their original cage for the remainder of their pregnancy. Females who had been placed with males were weighed every 2 to 3 d after mating until either E11.5 or E17.5. Successful attainment of gestation was determined by a weight gain > 2 g from E1.5 to E10.5. Pregnant dams were euthanized by cervical dislocation to prevent possible effects of CO_2_ on maternal physiology (73). Fetal sex was determined using real-time PCR for the Y chromosome and DNA isolated from fetal heads (E11.5) or tails (E17.5) (Transnetyx; Cordova, TN).

### Conventionalization of GF Mice.

CONV-D mice were generated by oral gavage of adult nonpregnant GF female mice with clarified cecal contents. The combined cecal contents of three nonpregnant CONV-R females was diluted 1/10 (w/v) with PBS with 0.05% L-cysteine hydrochloride. After vortexing four times for 30 s/cycle to disrupt clumps, the sample was filtered through a 100 µm sterile filter (Fisher Scientific; #08-771-19) and combined with equal volumes of sterile reduced PBS/30% glycerol. Each GF female mouse received 200 μL of clarified cecal mixture through a single gastric gavage; at least 17 d were allowed to elapse after gavage before starting timed matings. The methods used for defining the absolute abundances of bacterial taxa and short chain fatty acids in cecal samples are described in the *SI Appendix*. The procedures for bulk RNA-seq of intestinal segments and bulk and snRNA-seq of placentas harvested from mice are also provided in the *SI Appendix*.

### Placental Histology and Histomorphometric Measurements.

Placentas and the attached deciduas of GF, CONV-R, and CONV-D dams were collected at E11.5 and E17.5, rinsed in PBS, and fixed for 48 h at 4 °C in 10% neutral buffered formalin. Samples were rehydrated in 70% ethanol and refrigerated until processing for paraffin embedding. Serial 10 μm sections were generated. Sections were stained with hematoxylin (Vector, #H-3401) and eosin (Sigma-Aldrich, #HT110132) following standard procedures. Placental sections were scanned with a Hamamatsu NanoZoomer (HT) at 20X resolution.

LZ/JZ areas and vascular measurements were performed using QuPath v0.4.4. Placental zones were manually drawn and annotated. The method used for blood vessel histomorphometry in hematoxylin and eosin stained tissue sections was adapted from ref. 74. Vasculature in both the LZ and JZ were assumed to be oval-like in shape and the major/minor axes of the lumen were measured to calculate luminal area. Two measurements of endothelial cell and smooth muscle were averaged per vessel to determine perivascular thickness. Histomorphometric data were evaluated in a blinded fashion. The methods used for immunohistochemical analysis of paraffin-embedded sections are described in the *SI Appendix*. The procedures for placental homogenate assays and flow cytometry of decidua are also provided in the *SI Appendix*.

### Statistical Analysis.

Statistical significance was assigned to *P*-values < 0.05 using either nonparametric Wilcoxon test for maternal measurements and generalized linear mixed models for measurements involving multiple litter members to account for litter effects, using the lmerTest package in R. Unless otherwise noted, the BH false discovery method was used when accounting for multiple comparisons. Tests, the number of animals analyzed, and mean values and SD, and statistical comparison groups are indicated in the Figures and Figure legends.

## Supplementary Material

Appendix 01 (PDF)

Dataset S01 (XLSX)

Dataset S02 (XLSX)

Dataset S03 (XLSX)

Dataset S04 (XLSX)

Dataset S05 (XLSX)

Dataset S06 (XLSX)

Dataset S07 (XLSX)

Dataset S08 (XLSX)

Dataset S09 (XLSX)

## Data Availability

Shotgun DNA sequencing, bulk RNA-seq, and snRNA-seq datasets have been deposited in NCBI’s Sequence Read Archive (SRA) under project number PRJNA1284669 (75).
