# Supplementary material for: Effects of the gut microbiota on placental angiogenesis and intrauterine growth in gnotobiotic mice
Source: Proc Natl Acad Sci U S A. 2025 Jul 25;122(30):e2426341122. doi: 10.1073/pnas.2426341122 (PMC12318179; doi:10.1073/pnas.2426341122)
Supplement: Supplementary file 1 — Appendix 01 (PDF) [file pnas.2426341122.sapp.pdf]

PNAS

Supporting Information for

Effects of the gut microbiota on placental angiogenesis and intrauterine growth in gnotobiotic mice

Reyan Coskun[a,b,1], ZeNan L. Chang[a.b.c.1], Athziri Marcial Rodríguez[a.b], Haoxin Liu[a,b], Jiye Cheng[a,b], Yael Alippe[c], Michael S. Diamond[c.d.e], and Jeffrey I. Gordon[a,b,c,2]

[a]The Edison Family Center for Genome Sciences and Systems Biology, Washington University School of Medicine, St. Louis, MO 63110

[b]The Newman Center for Gut Microbiome and Nutrition Research, Washington University School of Medicine, St. Louis, MO 63110

[c]Department of Medicine, Washington University School of Medicine, St. Louis, MO 63110

[d]Department of Pathology and Immunology, Washington University School of Medicine, St. Louis, MO 63110

[e]Department of Molecular Microbiology, Washington University School of Medicine, St. Louis, MO 63110

[1]R.C. and Z.L.C. contributed equally to this work.

[2]To whom correspondence may be addressed. Email: jgordon@wustl.edu

This PDF file includes:

Figures S1 to S9
Tables S1 to S2
Legends for Datasets S1 to S9
Supporting text for Materials and Methods
SI References

Other supporting materials for this manuscript include the following:

Datasets S1 to S9

Supplementary Figures

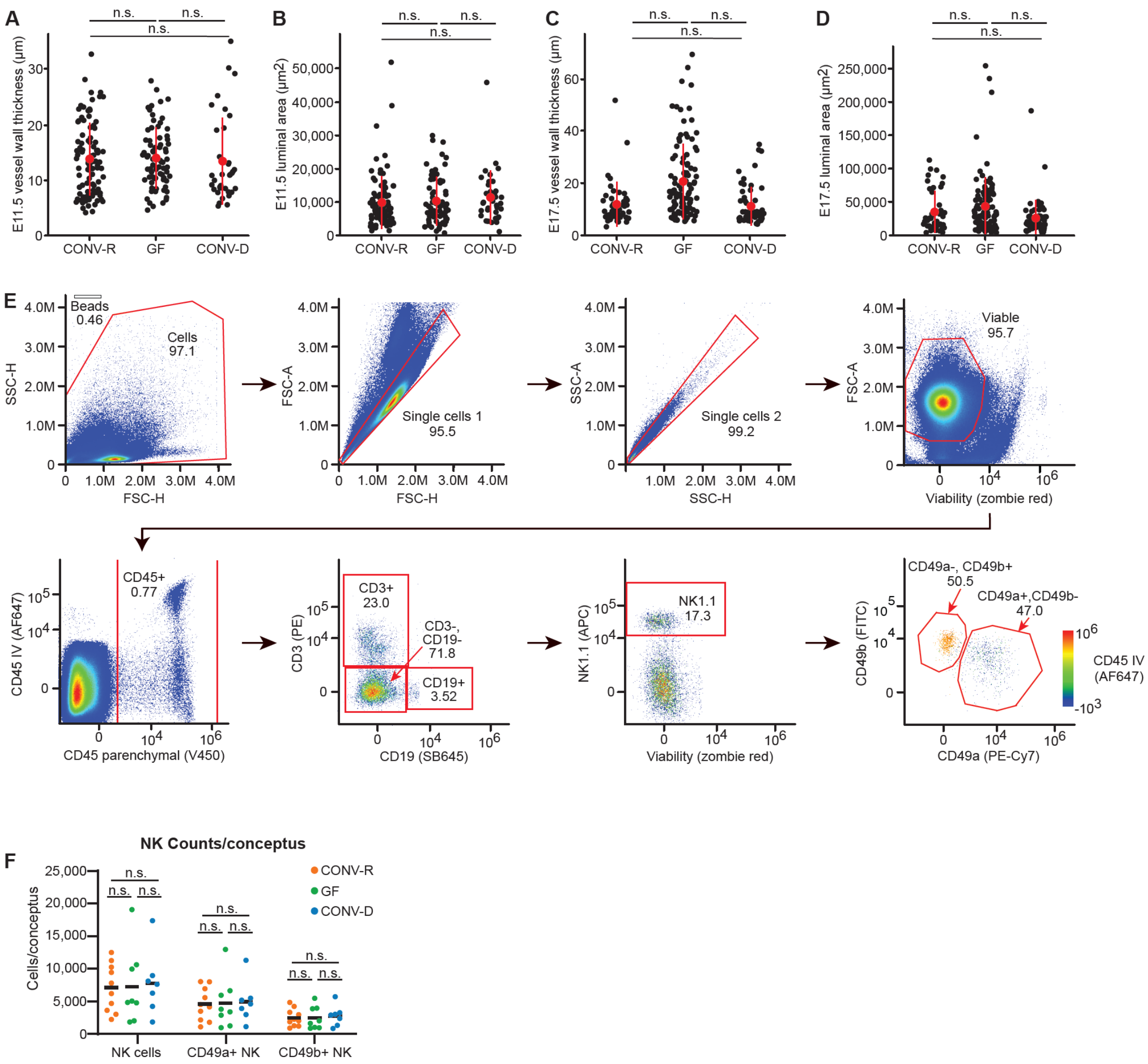

Fig. S1: Blood vessel histomorphometrics and uterine NK cell enumeration in the maternal decidua. (*A* and *B*) Histomorphometric measurements of (*A*) arterial wall thickness and (*B*) luminal area of spiral arteries in the maternal-derived decidua at E11.5 (n = 1-2 placentas/litter, 6 litters/treatment group). Adjusted *P*-values were defined using the linear mixed model (*Feature ~ Microbiota + (1 | Litter ID))* for pairwise comparisons, with the Benjamini-Hochberg (BH) correction applied. Mean values ± standard deviations are shown. (*C* and *D*) Measurements and analysis as in (*A*) and (*B*), except at E17.5 (n = 1-2 placentas, representing 4-5 litters/treatment group). Means ± standard deviations are shown. (*E*) Gating scheme for identifying uNK cells (and CD49a^{+} and CD49b^{+} subsets) in E11.5 deciduas by flow cytometry. All deciduas in each litter were pooled. (*F*) The number of decidual uNK cells per conceptus was measured at E11.5 in CONV-R, GF, and CONV-D dams (n = 7-10 dams/treatment group, Mann–Whitney–Wilcoxon test). Means are shown.

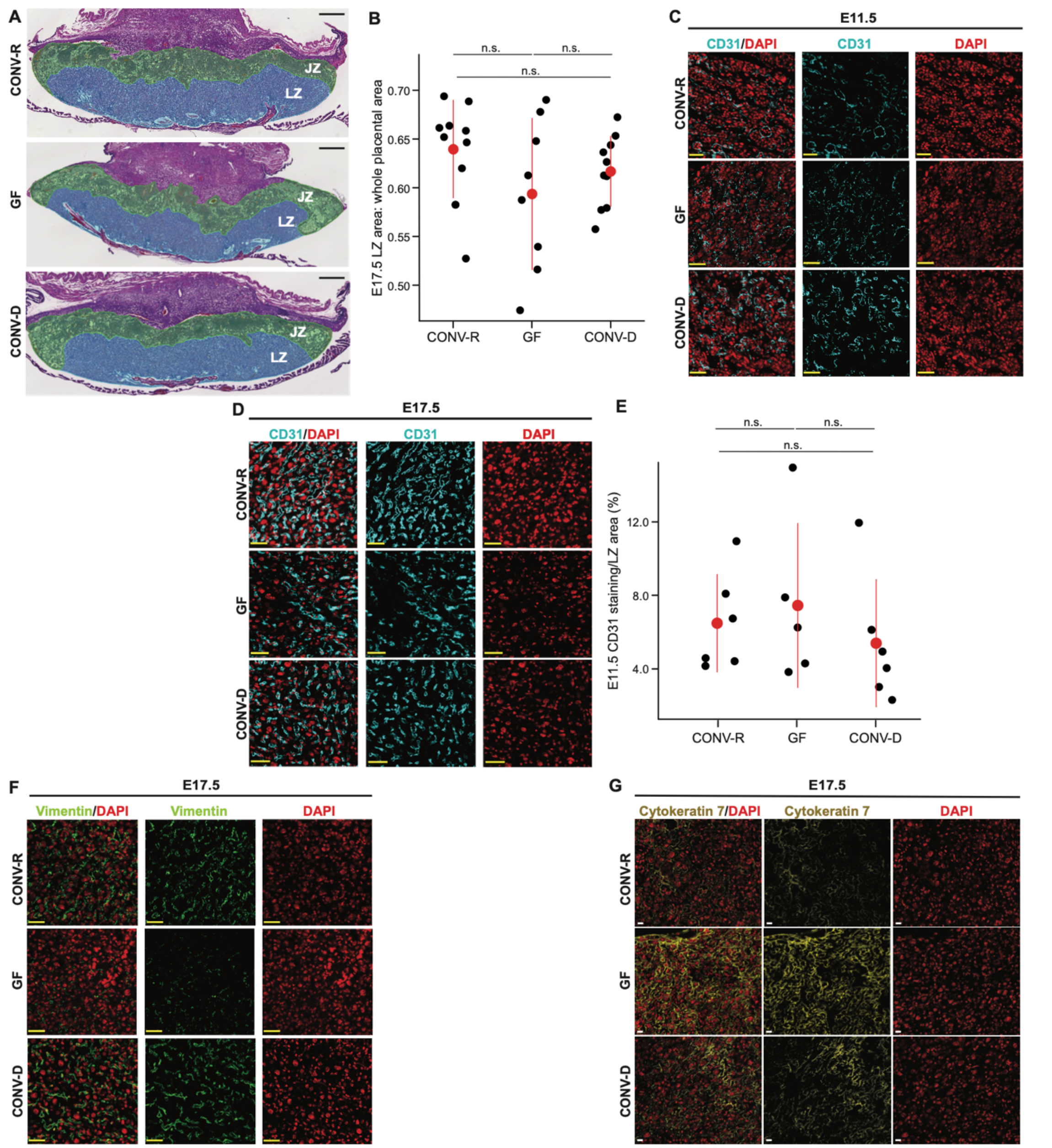

Fig. S2: LZ area and CD31, vimentin, and cytokeratin 7 immunostaining in CONV-R, GF and CONV-D dams. (*A*) LZ (blue) and JZ (green) in example cross sections of E17.5 placentas. (Scale bar, 500 µm.) (*B*) The ratio of LZ area to fetal placental (LZ + JZ) area (n = 1-2 placentas/litter, 5 litters/treatment group). Adjusted *P*-values were defined using a linear mixed model (*Ratio ~ Microbiota + (1 | Litter ID))* for pairwise comparisons, with the BH correction applied. Mean values ± standard deviations are shown. (*C* and *D*) Representative immunostaining of CD31 in (*C*) E11.5 LZ (Scale bar, 50 µm) and (*D*) E17.5 LZ (Scale bar, 50 µm). (*E*) The proportion of CD31-stained area in the LZ at E11.5 across the different groups (n = 1 section/placenta, 1-2 placentas/litter, 4-6 litters/treatment group). Adjusted *P*-values were defined using a linear mixed model (*% Staining ~ Microbiota + (1 | Litter ID))* for pairwise comparisons, with the BH correction applied. Mean values ± standard deviations are shown. (*F*) Representative immunostaining of vimentin in E17.5 LZ. (Scale bar, 50 µm.) (*G*) Representative immunostaining of cytokeratin 7 in E17.5 LZ (n = 1 section/placenta, 1 placenta/litter, 3 litters/treatment group). (Scale bar, 20 µm.)

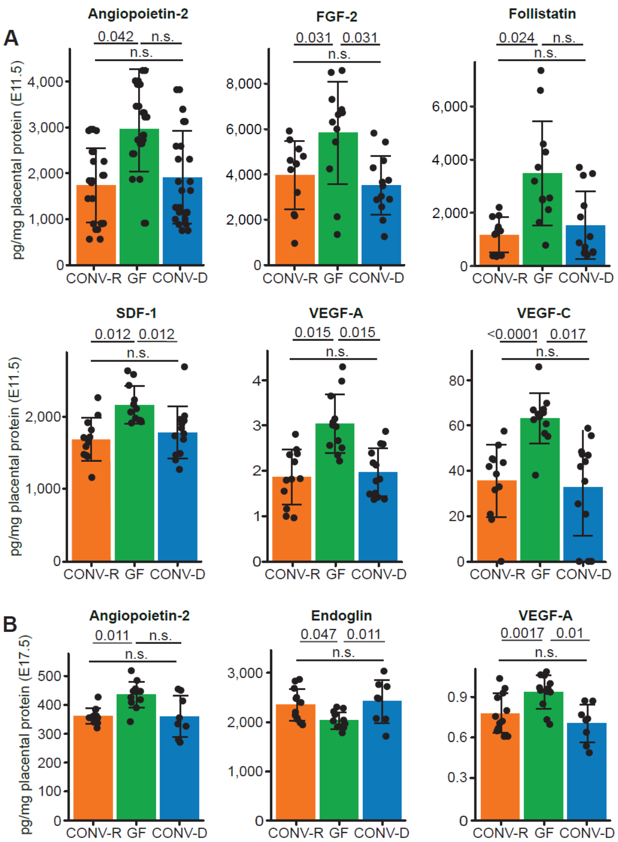

Fig. S3. Quantification of angiogenesis-associated proteins in CONV-R, GF, and CONV-D placentas. (*A*) The angiogenesis-associated proteins angiopoietin-2, FGF-2, follistatin, SDF-1, VEGF-A, and VEGF-C were quantified in placental homogenates at E11.5 (n = 4 dams/group, 3-4 placentas/dam). Adjusted *P*-values are shown as calculated using a linear mixed model (*Protein level ~ Microbiota + (1 | Litter ID)*) for pairwise comparisons with the BH correction applied. Means ± standard deviations are shown. (*B*) Quantification as in panel A but at E17.5. Means± standard deviations are shown.

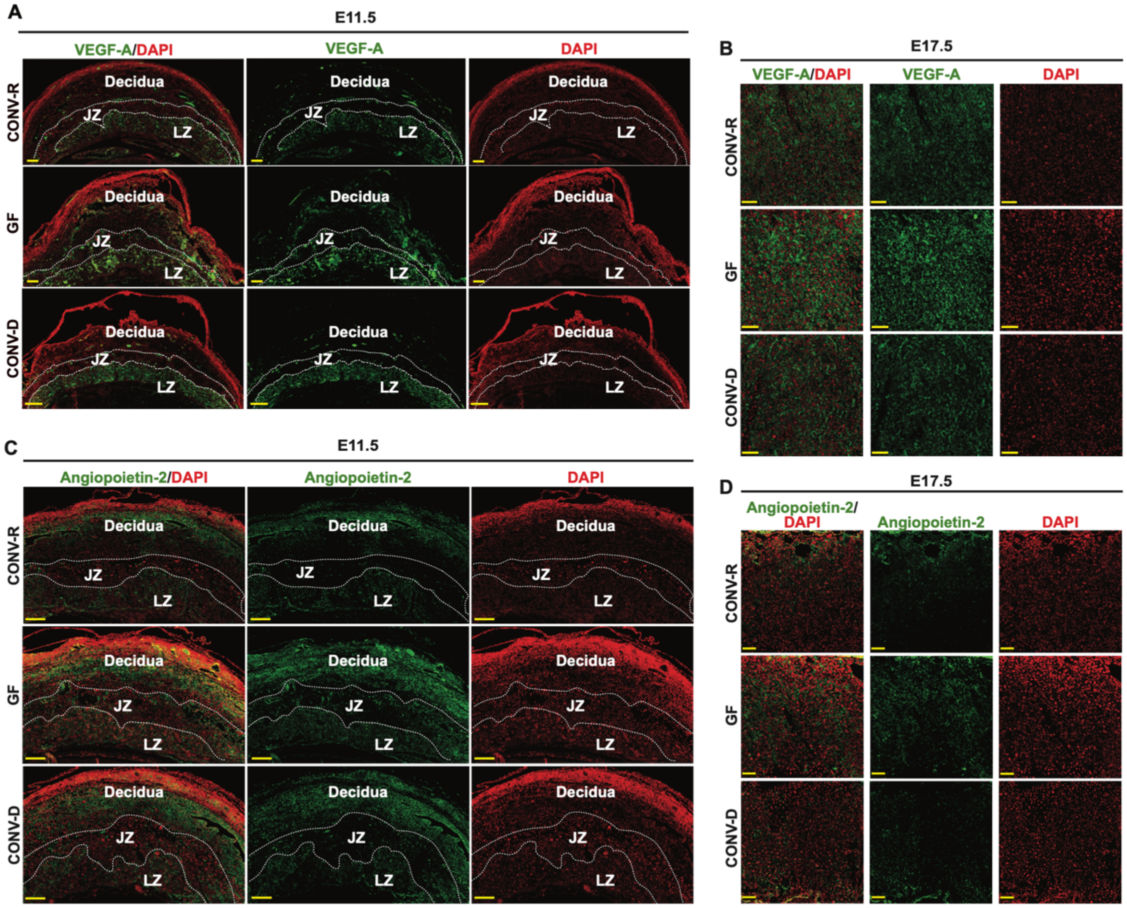

Fig. S4: Immunostaining for VEGF-A and angiopoietin-2 in E11.5 and E17.5 placentas harvested from CONV-R, GF and CONV-D dams. Representative sections of (*A*) E11.5 and (*B*) E17.5 placenta immunostained for VEGF-A (n = 1 placenta/litter, 3 litters/treatment group). Scale bars are 400 µm for E11.5 images and 100 µm for E17.5 images. Only LZ shown in (*B*). Sections of (*C*) E11.5 and (*D*) E17.5 placenta immunostained for angiopoietin-2 (n = 1 placenta/litter, 3 litters/treatment group). Scale bars are 100 µm for E11.5 images and 250 µm for E17.5 images. Only LZ shown in (*D*).

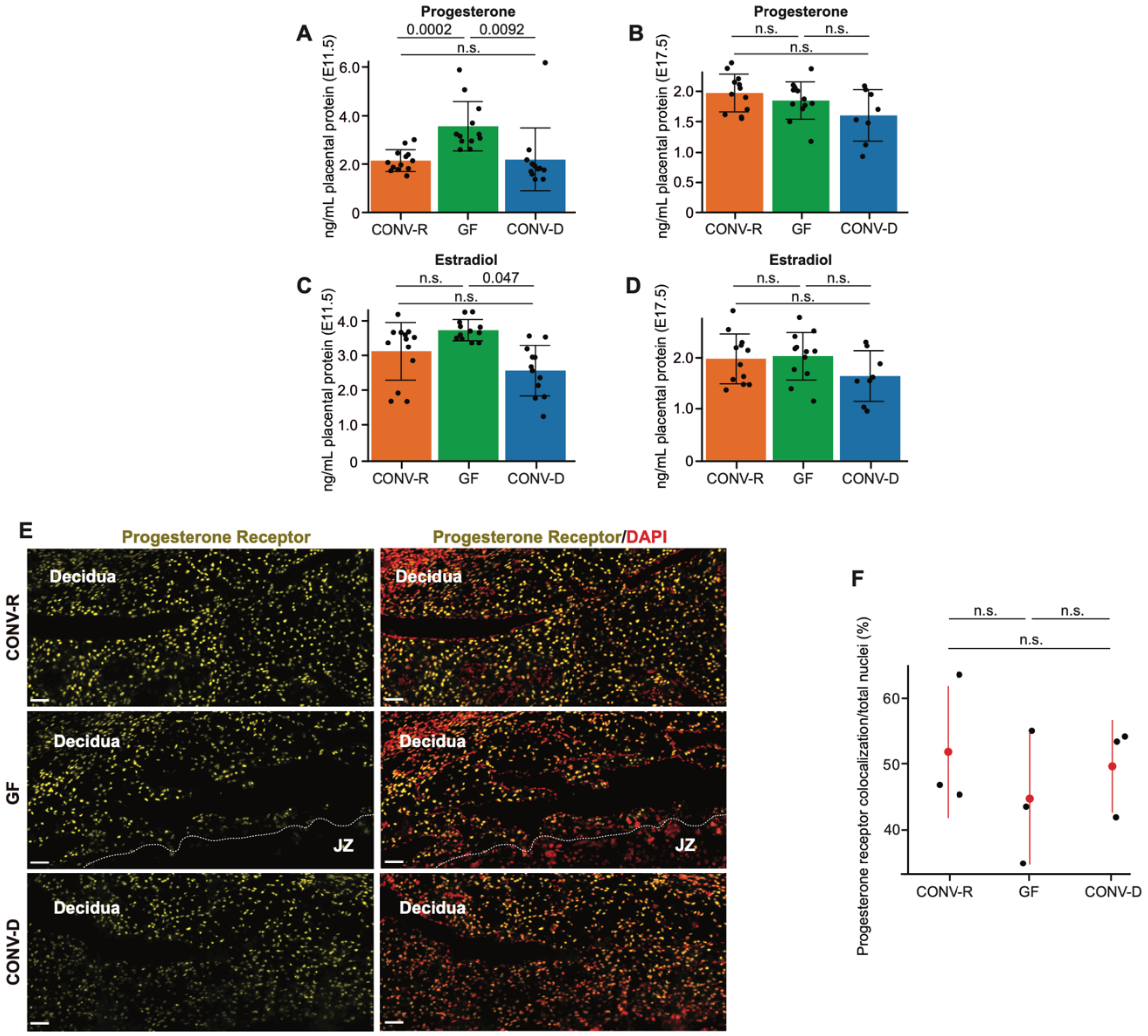

Fig. S5: Quantification of progesterone and estradiol in CONV-R, GF, and CONV-D placentas. (*A* to *D*) Estradiol and progesterone levels quantified in placental homogenate at E11.5 and E17.5 across treatment groups (n = 4 dams/treatment group, 3-4 placentas/dam). Statistically significant adjusted *P*-values are shown, calculated with a linear mixed model (*Hormone level ~ Microbiota + (1 | Litter ID)*) for pairwise comparisons, with the BH correction applied. Means ± standard deviations are shown. (*E*) Representative immunostaining of the progesterone receptor at E11.5 placental cross-sections (n =1 placenta/litter, 3 litters/treatment group). (Scale bars, 50 μm.) (*F*) Proportion of nuclei with progesterone receptor co-localization. Adjusted *P*-values were calculated using a linear mixed model (*% Progesterone Receptor-Nuclei Co-localization ~ Microbiota + (1 | Litter ID))* for pairwise comparisons, with the BH correction applied. Means ± standard deviations are shown.

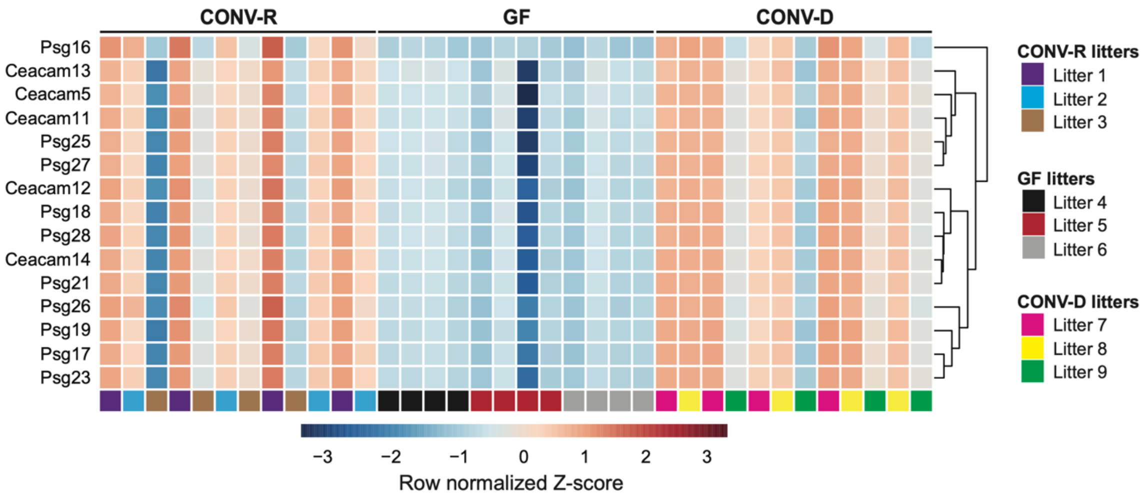

Fig. S6: Expression of glycoprotein transcripts in E11.5 fetal placenta. Pregnancy-specific glycoproteins are reported to be reduced in cases of reduced fetal growth; the expression of glycoproteins including *Psgs* in the bulk RNA-seq dataset are presented as a heatmap (n = 3 litters/treatment group, 4 placentas/litter, data normalized and compared using DESeq2).

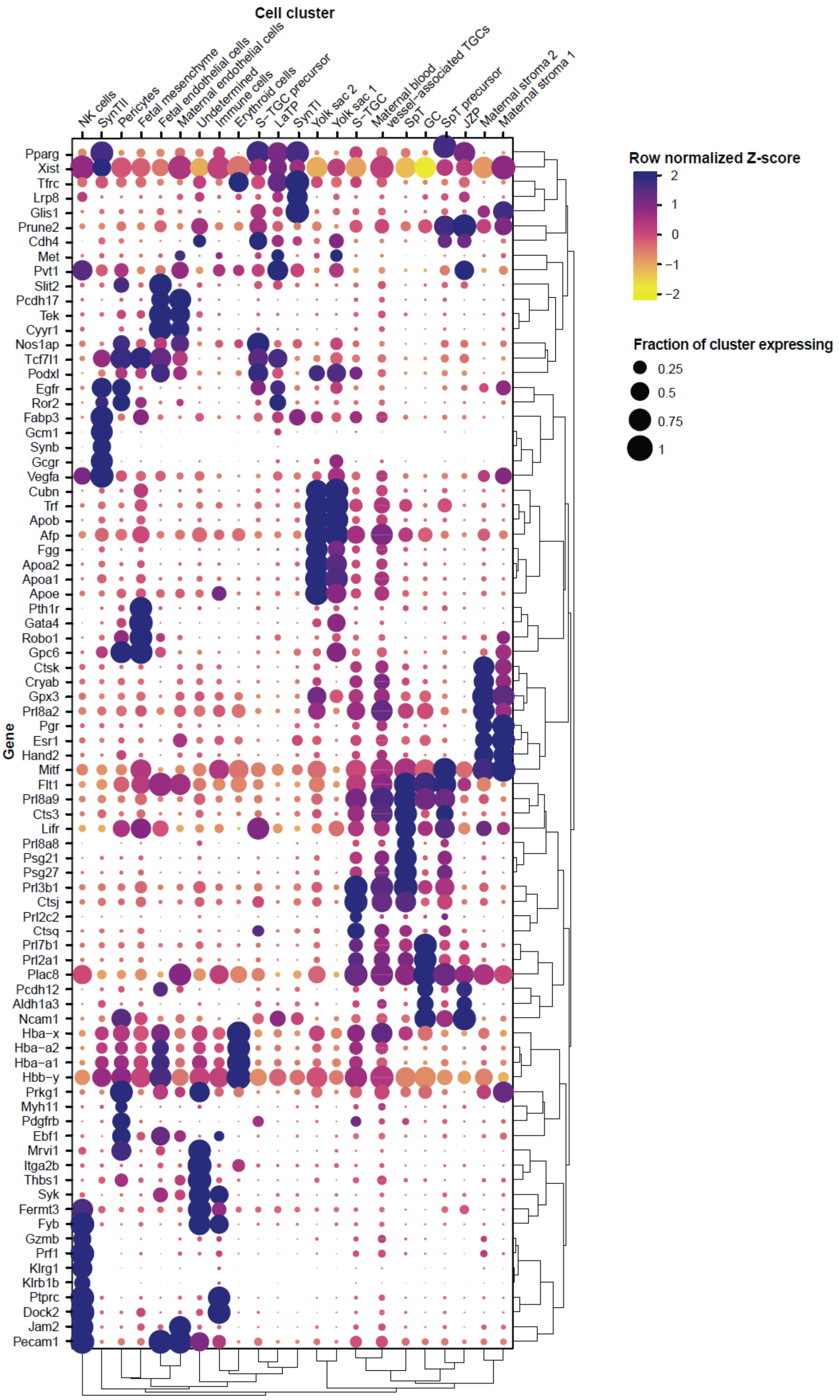
Cell cluster
NK cells
SynTII
Pericytes
Fetal mesenchyme
Fetal endothelial cells
Maternal endothelial cells
Undetermined
Immune cells
Erythroid cells
S-TGC precursor
LaTP
SynTI
Yolk sac 2
Yolk sac 1
S-TGC
Maternal blood
vessel-associated TGCs
SpT
GC
SpT precursor
JZP
Maternal stroma 2
Maternal stroma 1
Gene
Pparg
Xist
Tfrc
Lrp8
Glis1
Prune2
Cdh4
Met
Pvt1
Slit2
Pcdh17
Tek
Cyyr1
Nos1ap
Tcf7l1
Podxl
Egfr
Ror2
Fabp3
Gcm1
Synb
Gcgr
Vegfa
Cubn
Trf
Apob
Afp
Fgg
Apoa2
Apoa1
Apoe
Pth1r
Gata4
Robo1
Gpc6
Ctsk
Cryab
Gpx3
Prl8a2
Pgr
Esr1
Hand2
Mitf
Flt1
Prl8a9
Cts3
Lifr
Prl8a8
Psg21
Psg27
Prl3b1
Ctsj
Prl2c2
Ctsq
Prl7b1
Prl2a1
Plac8
Pcdh12
Aldh1a3
Ncam1
Hba-x
Hba-a2
Hba-a1
Hbb-y
Prkg1
Myh11
Pdgfrb
Ebf1
Mrvi1
Itga2b
Thbs1
Syk
Fermt3
Fyb
Gzmb
Prf1
Klrg1
Klrb1b
Ptprc
Dock2
Jam2
Pecam1
Row normalized Z-score
2
1
0
−1
−2
Fraction of cluster expressing
0.25
0.5
0.75
1

Fig. S7: Marker genes used to identify cell clusters in snRNA-seq datasets generated from E11.5 placentas. CellRanger, CellBender, then the Seurat framework were used to identify 32,571 high quality nuclei that were then clustered by the *FindNeighbors* and *FindClusters* functions (see Supplementary *Materials and Methods*). Marker genes collected by literature review of prior snRNA-seq results (see supplementary refs. 1-4) were used to assign clusters to 22 cell types. A dot-plot of marker-gene expression across the cell-type assignments is shown.

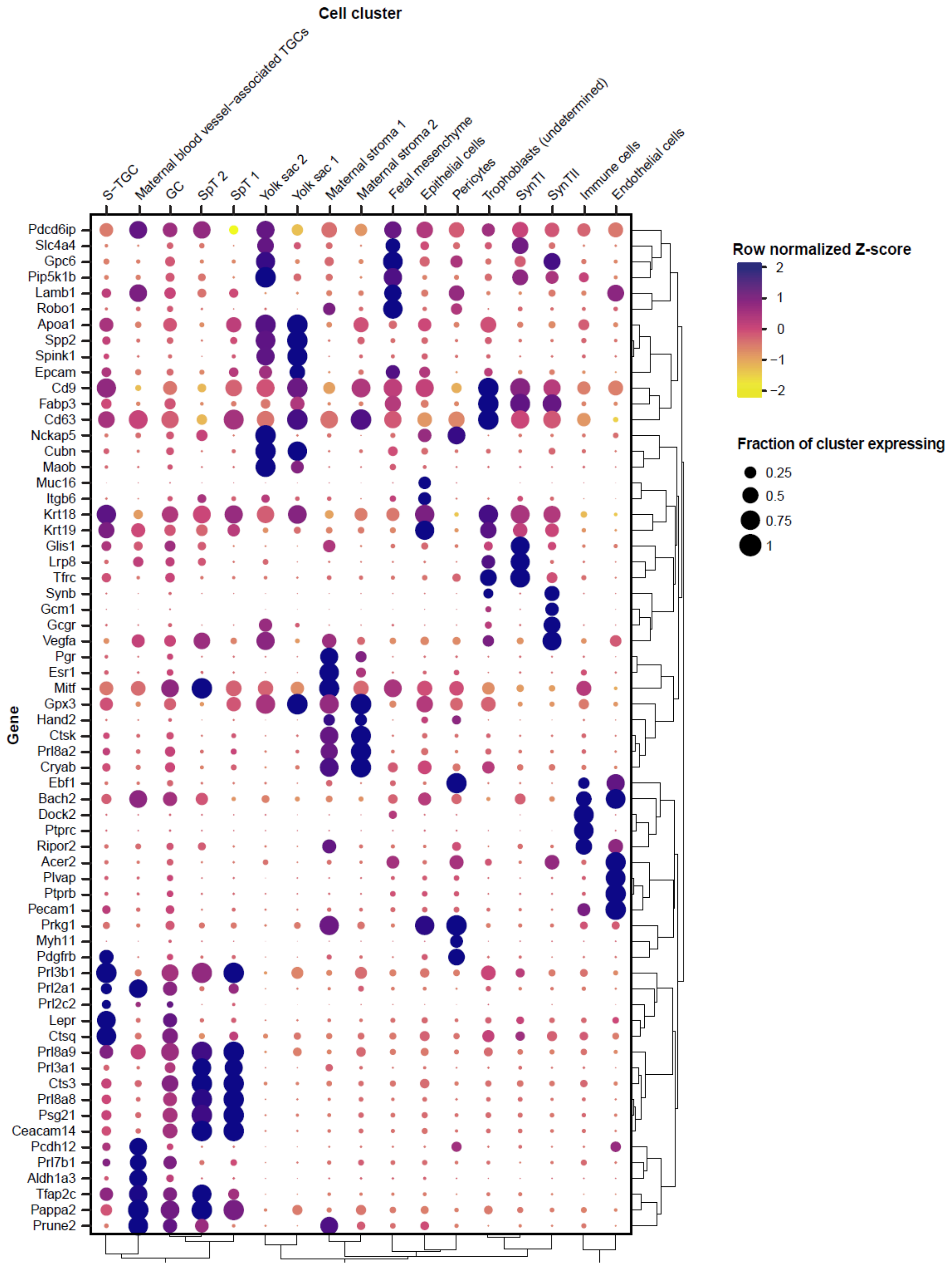

Fig. S8: Marker genes used to identify cell clusters in snRNA-seq datasets generated from E17.5 placentas. CellRanger, CellBender, then the Seurat framework were used to identify 36,918 high quality nuclei that were then clustered by the *FindNeighbors* and *FindClusters* functions (see Supplementary *Materials and Methods*). Marker genes collected by literature

review of prior snRNA-seq results (see supplementary refs. 1-4) were used to assign clusters to 17 cell types. A dot-plot of marker-gene expression across the cell-type assignments is shown.

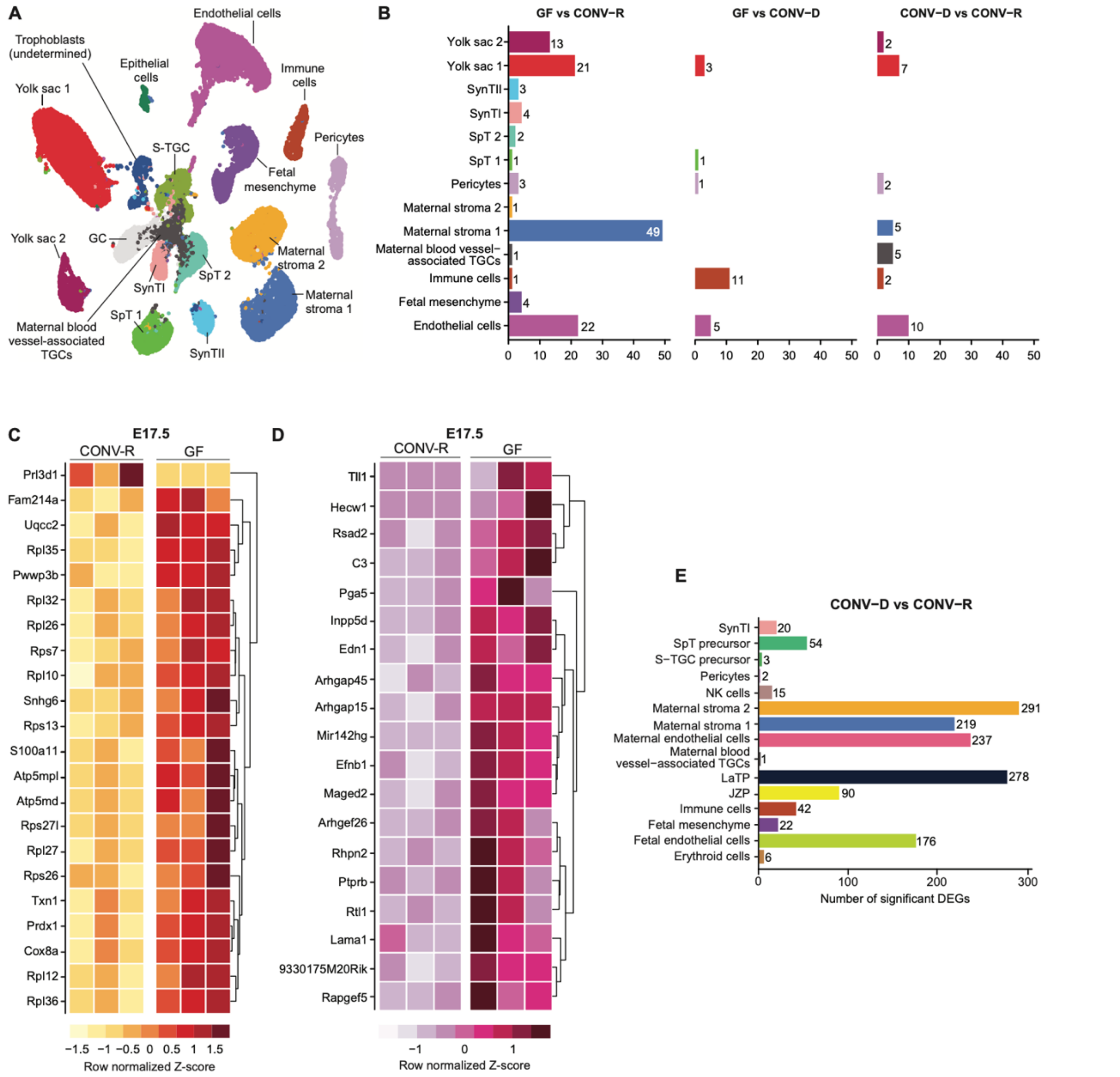

Fig. S9: snRNA-seq of E17.5 CONV-R, GF, and CONV-D placentas and number of DEGs in E11.5 CONV-R, GF, and CONV-D pseudobulk analysis. (*A*) UMAP representation of 36,918 nuclei across nine E17.5 placentas (3 CONV-R, 3 GF, and 3 CONV-D) clustered and assigned to 17 cell types. (*B*) Number of DEGs identified in each cell type by pseudobulk analysis of E17.5 GF versus CONV-R or CONV-D and CONV-D versus CONV-R placentas (DESeq2, $|\log_2(\text{fold-difference})| > 0.5$, adjusted *P*-value < 0.05, Wald test with BH correction). (*C*) DEGs identified when comparing GF to CONV-R endothelial cells ($|\log_2(\text{fold-difference})| > 0.7$). (*D*) DEGs identified when comparing GF to CONV-R maternal stroma 1 cells ($\log_2(\text{fold-difference}) > 2.5$). (*E*) Number of DEGs in each cell type identified by pseudobulk analysis of E11.5 CONV-D versus CONV-R placentas (DESeq2, $|\log_2(\text{fold-difference})| > 0.5$).

Supplementary Tables

Reagent	Type	Host and/or Fluorophore	Dilution (µg/mL)	Vendor	Catalog no.
CD31	1°	Rabbit	2.1	Abcam	ab182981
Vimentin	1°	Rabbit	1.3	Abcam	ab92547
Cytokeratin 7	1°	Rabbit	7.7	Abcam	ab181598
VEGF-A	1°	Mouse	5.0	Abcam	ab1316
Angiopoietin-2	1°	Rabbit	5.0	Invitrogen	PIMA532759
Progesterone Receptor	1°	Rabbit	0.6	Abcam	ab101688
Phospho-VEGFR2 (Tyr1175)	1°	Rabbit	10	Invitrogen	PA5-105167
Phospho-MAPK (p38)	1°	Rabbit	0.7	Invitrogen	MA5-15177
Phospho-ERK1/2 (p44/42)	1°	Rabbit	2.5	Cell Signaling Technology	4370S
Alexa Fluor 546 Anti-Rabbit IgG H+L, Highly Cross-Absorbed	2°	Goat, Alexa Fluor 546	2	Invitrogen	A11035
Alexa Fluor 647 Anti-Rabbit IgG (H+L), Highly Cross-Absorbed	2°	Goat, Alexa Fluor 647	2	Invitrogen	A21245
TRITC Anti-Mouse IgG H&L, Preabsorbed	2°	Goat, TRITC	2	Abcam	ab7065
Alexa Fluor 488 Anti-Rabbit IgG H&L	2°	Goat, Alexa Fluor 488	2	Abcam	ab150077

Table S1. Reagents used for immunohistochemical analyses

Anti-Mouse Antibody	Host	Fluorophore	Clone	Vendor	Dilution	Catalog no.
CD49a	Armenian Hamster	PE-Cy7	HMa1	Biolegend	1/200	142607
CD45 [parenchymal]	Rat	V450	30-F11	BD Horizon	1/200	560501
CD45 [IV]	Rat	AF647	S18009D	Biolegend	IV injection: 7/40	160304
CD3	Rat	PE	17A2	BD Biosciences	1/100	555275
NK1.1	Mouse	APC	PK146	Biolegend	1/200	108710
CD49b	Armenian Hamster	FITC	HMa2	Biolegend	3/200	103504
CD19	Rat	SB645	eBio1D3 (1D3)	Invitrogen	1/200	64-0193-82

Table S2. Antibodies employed for flow cytometry

Supplementary Dataset Legends

Dataset S1: Fetal weights, placental weights, and litter sizes at E11.5 and E17.5 across microbiota groups. (*A*) Fetal and placental weights at E11.5 and E17.5 across treatment groups, separated by fetal sex. Pairs of treatment groups were compared by linear mixed models, with the BH method used to correct for multiple comparisons. Linear mixed models: *Fetal weight ~ Microbiota + Litter Size + (1 | Litter ID)* (CONV-R: 11 dams, 34 female fetuses, 36 male fetuses; GF: 14 dams, 32 female fetuses, 43 male fetuses; CONV-D: 12 dams, 27 female fetuses, 47 male fetuses at E11.5; and CONVR: 6 dams, 23 female fetuses, 23 male fetuses; GF: 5 dams, 17 female fetuses, 13 male fetuses; CONV-D: 7 dams, 25 female fetuses, 28 male fetuses at E17.5); *Placental weight ~ Microbiota + Litter Size + (1 | Litter ID)* (CONV-R: 11 dams, 34 placentas, 36 male placentas; GF: 14 dams, 32 female placentas, 44 male placentas; CONV-D: 12 dams, 27 female placentas, 46 male placentas at E11.5**;** and CONV-R: 6 dams, 20 female placentas, 20 male placentas; GF: 5 dams, 16 female placentas, 12 male placentas; CONV-D: 7 dams, 22 female placentas, 18 male placentas at E17.5). (*B*) Litter sizes across treatment groups assessed at E11.5 or E17.5. Statistical significance was determined using the Mann–Whitney–Wilcoxon test and the BH correction for multiple comparisons (CONV-R: 15 litters at E11.5, 8 litters at E17.5; GF: 15 litters at E11.5, 8 litters at E17.5; CONV-D: 12 litters at E11.5, 8 litters at E17.5). In each table, means and standard deviations are shown at the bottom.

Dataset S2: V4-16S rRNA sequencing across nonpregnant, E11.5, and E17.5 CONV-R mice. (*A*) Taxonomic assignments and absolute abundances of amplicon sequence variants (ASVs). N.D., not detected. (*B*) Alpha diversity metrics across pregnancy stages. Mann–Whitney–Wilcoxon test with the BH correction was used to test for differences across pregnancy within each microbiota group. (*C*) Absolute and relative abundances of taxa at various levels of taxonomic resolution.

Dataset S3: Differential expression analysis using bulk RNA-seq to compare different gut segments of E11.5 or E17.5 mice to their nonpregnant CONV-R and GF counterparts along the length of the gut. (*A* and *B*) DESeq2 was used to identify DEGs in duodenal, jejunal, ileal, and colonic segments at E11.5 in comparison to nonpregnant counterparts (*A*), and at E17.5 in comparison to nonpregnant counterparts (*B*) (n = 4-5/treatment group). DEGs were defined as having a BH adjusted *P*-value < 0.05 and fold-difference > 1.5. (*C* and *D*) Using DESeq2 results to rank genes, GSEA was performed across GO-BP gene sets with a focus on leading edges containing DEGs in duodenal, jejunal, ileal, and colonic intestinal segments harvested from CONV-R or GF mice at E11.5 in comparison to nonpregnant counterparts (*C*), and at E17.5 in comparison to nonpregnant counterparts (*D*). Statistical significance was defined as a BH adjusted *P*-value < 0.05. (*E*) Significantly enriched GO-BP terms shown in (*C*) and (*D*) that are related to blood vessel formation.

Dataset S4: Bulk RNA-seq to identify differential gene expression comparing GF to CONV-R dams at different stages of pregnancy along the length of their intestines. (*A* to *D*) DESeq2 was used to identify DEGs in GF versus CONV-R mice who are nonpregnant, at E11.5 or at E17.5. Duodenum (*A*). Jejunum (*B*). Ileum (*C*). Colon (*D*) (n = 4-5/treatment group). DEGs were defined as having a BH adjusted *P*-value < 0.05 and fold-difference > 1.5. (*E* to *H*) Using DESeq2 results to rank genes, GSEA was performed across GO-BP gene sets to identify statistically significant gene set enrichments with a focus on leading edges containing DEGs in GF versus CONV-R mice who are nonpregnant, at E11.5, or at E17.5. Duodenum (*E*). Jejunum (*F*). Ileum (*G*). Colon (*H*). Statistical significance was defined as a BH adjusted *P*-value < 0.05. (*I*) Significantly enriched GO-BP terms shown in panels *E* to *H* that are related to blood vessel formation.

Dataset S5: Levels of placental angiogenic proteins and their correlations with fetal and/or placental weights at E11.5 or E17.5. (*A*) Angiogenesis-associated proteins quantified by Luminex assay in fetal placental homogenates at E11.5 and E17.5 (n = 4 dams/group, 3-4 placentas/dam). Statistically significant adjusted *P*-values are shown as calculated using a linear mixed model (*Protein level ~ Microbiota + (1 | Litter ID)*) for pairwise comparisons with the BH

correction applied. (*B*) For proteins with at least one statistically significant difference between groups, a Spearman's correlation of their levels with fetal and placental weights was performed within each treatment group at E11.5 or E17.5. The BH correction for multiple comparisons was used to calculate *P*-values.

Dataset S6: Quantification of short chain fatty acids in cecal contents from CONV-R, GF, and CONV-D mice at different stages of pregnancy. GC-MS was used to quantify levels of the indicated SCFAs from homogenized cecal contents collected from nonpregnant and pregnant CONV-R, GF, and CONV-D dams (n = 6-8, 4-6, and 5-9/treatment group respectively). Mann–Whitney–Wilcoxon test with the BH correction was used to test for differences across pregnancy within each microbiota group.

Dataset S7: Identification of differential gene expression using bulk RNA-seq to compare the fetal compartments of GF E11.5 placentas to CONV-R or CONV-D E11.5 placentas. (*A*) DESeq2 was used to identify statistically significant differentially expressed genes in GF versus CONV-R and GF versus CONV-D E11.5 placentas (n = 4 dams/treatment group with 4 placentas /dam). Statistical significance was defined as an adjusted *P*-value < 0.05 and fold-difference > 1.5. (*B*) Using DESeq2 results to rank genes, GSEA was performed across the Hallmark and GO gene sets. Statistical significance was defined as having an BH adjusted *P*-value < 0.05.

Dataset S8: snRNA-seq of E11.5 placentas. snRNA-seq was performed on 10 E11.5 placentas (3 CONV-R, 3 GF, and 4 CONV-D). (*A*) The *FindMarkers* function in the Seurat package identified genes that were most specific to each cluster to assist in cell cluster assignments. (*B*) Pseudobulk analysis with DESeq2 was used to identify statistically significant DEGs for each cell cluster and each comparison (GF versus CONV-R or CONV-D, CONV-D versus CONV-R). Significant DEGs were defined by an adjusted *P*-value < 0.05 and $|\log_2(\text{fold-difference})| > 0.5$. (*C*) Full gene names, gene functions (as per NIH NLM NCBI Gene annotations), and subcellular compartments of the gene products (NIH NLM NCBI Gene annotations) are tabulated for DEGs in the comparison of GF versus CONV-R maternal endothelial cells. (*D*) Using DESeq2 analysis of GF versus CONV-R maternal endothelial cells to rank genes, GSEA was performed across the Hallmark and GO gene sets. Statistical significance was defined by a BH adjusted *P*-value < 0.05. (*E*) Gene annotations as in (*C*), except for DEGs in the comparison of endothelial cells in the fetal compartments of GF versus CONV-R placentas. (*F*) An analysis comparable to that in (*D*) but in this case involving a comparison of GF versus CONV-R fetal endothelial cells. Statistical significance was defined as a BH adjusted *P*-value < 0.05. (*G*) Gene annotations as in (*C*), except for DEGs in GF versus CONV-D maternal stroma 2 cells. (*H*) An analysis comparable to that in (*D*) but in this case involving a comparison of GF versus CONV-D maternal stroma 2 cells. Statistical significance defined as a BH adjusted *P*-value < 0.05. (*I*) An analysis comparable to that in (*D*) but in this case involving a comparison of CONV-D versus CONV-R fetal endothelial cells. Statistical significance defined as a BH adjusted *P*-value < 0.05. (*J*) An analysis comparable to that in (*D*) but in this case involving a comparison of CONV-D versus CONV-R maternal endothelial cells. Statistical significance defined as a BH adjusted *P*-value < 0.05.

Dataset S9: snRNA-seq of E17.5 placentas. snRNA-seq was performed using nine E17.5 placentas (3 CONV-R, 3 GF, and 3 CONV-D). (*A*) *FindMarkers* function in the Seurat package was used to identify genes most specific to each cluster to assist in cell type assignments. (*B*) Pseudobulk analysis with DESeq2 used to identify statistically significant DEGs in each cell cluster and each comparison type (GF versus CONV-R or CONV-D, CONV-D versus CONV-R). Significant DEGs defined by an adjusted *P*-value < 0.05 and $|\log_2(\text{fold-difference})| > 0.5$. (*C*) The full gene names, gene functions (as per NIH Gene annotations), and subcellular compartments of the gene products (NIH NLM NCBI Gene annotations) tabulated for DEGs in GF versus CONV-R fetal endothelial cells. (*D*) Gene annotations as in (*C*), except for DEGs in GF versus CONV-R maternal stroma 1 cells.

Supplementary Materials and Methods

V4-16S rRNA amplicon sequencing

Fecal samples were processed and analyzed as previously described (5). DNA was extracted from flash-frozen cecal contents by bead-beating with 250 µL of 0.1 mm zirconia/silica beads and one 3.97 steel ball in a solution containing 500 µL phenol:chloroform:isoamyl alcohol (25:24:1), 210 µL 20% SDS, and 500 µL buffer A (200 mM NaCl, 200 mM Trizma base, 20 mM EDTA), followed by purification (QiaQuick columns, Qiagen, #28104) and storage in Tris-EDTA buffer. Purified DNA was adjusted to a concentration of 2 ng/µL. PCR was used to generate V4-16S rRNA amplicons, and the amplicons sequenced with an Illumina MiSeq instrument [paired-end 250 nt reads, 63,547 ± 4813 reads (mean ± SD)/sample]. The ZymoBIOMICS Spike-in Control was utilized to define absolute abundances of taxa (Zymo Research, Irvine, CA). Sequencing reads were filtered and trimmed using BBMap (v38.63) and DADA2 (v1.26.0) (6). DADA2 was then employed to obtain and quantify error-corrected amplicon sequence variants (ASVs) in R (v4.1.1). Taxonomic assignments were performed using Ribosomal Database Project Naïve Bayesian Classifier (database v18) 80% (option 'minboot = 80'), with further species assignment if there was a unique sequence match. Tables of ASV abundances (counts) for each sample were combined with sample metadata and taxonomic assignment into a phyloseq (v1.3.0) object in R. Samples with fewer than 2000 reads were excluded from further analysis. Contaminating mitochondrial or chloroplast reads were removed, along with any bacterial ASVs lacking a phylum-level taxonomic classification. A filter was applied to remove any ASVs present at fewer than 5 counts in less than 5% of samples. Statistically significant differences in ASV relative and absolute abundances were assessed by applying linear models to log-transformed data using the MaAsLin2 framework and the Bonferroni correction to reduce false discovery rate in the setting of multiple comparisons (7).

Short chain fatty acid measurements

Targeted gas chromatography-mass spectroscopy (GC-MS) was used to quantify levels of short chain fatty acids. Aliquots of flash-frozen cecal contents were weighed and supplemented with 10 µL of a mixture of internal standards (20 mM of acetic acid-13C2,D4, propionic acid-D6, butyric acid-^{13}C4, lactic acid-3,3,3- D3, and succinic acid-^{13}C4). After addition of 20 µL of 33% HCl and 1 mL diethyl ether, the mixture was vortexed vigorously for 10 minutes and centrifuged (4,000 × g for 5 minutes). The upper organic layer was transferred to another vial and a second diethyl ether extraction was performed. After combining the two ether extracts, a 60 µL aliquot was removed, combined with 20 µL N-tert-butyldimethylsilyl-N-methyltrifluoroacetamide (MTBSTFA) in a GC auto-sampler vial with a 200 µL glass insert, and incubated for 2 h at room temperature. Derivatized samples (1 µL) were injected with 15:1 split into an Agilent 7890B/5977B GC-MS system. Quantification was performed by isotope dilution GC-MS using selected ion monitoring; the m/z for native and labeled molecular peaks were 117 and 122 (acetate), 131 and 136 (propionate), 145 and 149 (butyrate), 261 and 264 (lactate), and 289 and 293 (succinate), respectively.

Bulk RNA-seq

RNA was extracted from flash-frozen ~1 cm sections of duodenum, jejunum, ileum, or colon, or one-third of the fetal placenta, using the RNeasy 96 Kit (Qiagen). Total RNA quantification and quality assessment was performed using a TapeStation (Agilent). cDNA libraries were generated using the Illumina TruSeq Stranded Total RNA Prep with Ribo-Zero kit (Illumina). Barcoded libraries were sequenced on Illumina NovaSeq 6000 [150 nt pair-end reads to a depth of $3.62 \times 10^7 \pm 5.90 \times 10^6$ reads/sample (mean ± SD) for intestinal segments and $4.71 \times 10^7 \pm 4.96 \times 10^6$ reads/sample (mean ± SD) for fetal placental/decidua samples]. DESeq2-based differential gene expression analysis was performed where an adjusted *P*-value < 0.05 (Wald's test with the BH correction) along with a fold-difference of at least 1.5 was defined as significant (8). Genes were ranked by $-\log_{10}$(adjusted *P*-value)*$\log_2$(fold-difference) and then GSEA was performed using the fgsea R package (9). Mouse gene sets were selected from the MSigDB (10).

snRNA-seq

Placentas were dissected from the underlying decidual tissue at both E11.5 and E17.5. Two-thirds of the placenta by weight was used for nuclear extraction (n = 1 placenta from a given litter, n = 3 litters/treatment group). Using methods adapted from a recently described protocol for jejunal segments (11), placentas were thawed and minced in lysis buffer [25 mM citric acid, 250 mM sucrose, 0.1% NP-40, and 1X protease inhibitor (Roche)]. Nuclei were extracted via a Dounce homogenizer (Wheaton). E11.5 placentas were dounced 5 times with a loose pestle and 10 times with the tight pestle. E17.5 placentas were dounced 10 times with the loose pestle and 5 times with the tight pestle. Samples were then washed 3 times with buffer [25 mM citric acid, 0.25 M sucrose, 1X protease inhibitor (Roche)], and filtered successively through 100 µm, 70 µm, and 40 µm diameter strainers (pluriSelect) to obtain single nuclei in resuspension buffer [25 mM KCl, 3 mM MgCl2, 50 mM Tris, 1 mM DTT, 0.4 U/µL RNase inhibitor (Sigma) and 0.4 U/µL Superase inhibitor (ThermoFisher)]. Approximately 10,000 nuclei per sample were subjected to gel bead-in-emulsion (GEM) generation, reverse transcription, and construction of libraries for sequencing according manufacturer's instructions in the 3' gene expression v3.1 kit (10X Genomics). The concentration of the final cDNA libraries was determined by qPCR using the KAPA library Quantification Kit (KAPA Biosystems/Roche) to produce cluster counts appropriate for the Illumina NovaSeq6000 instrument. Normalized libraries were sequenced (NovaSeq6000 S4 Flow Cell using the XP workflow and a 50x10x16x150 sequencing recipe according to manufacturer protocol). A sequencing depth of 600 million read pairs was targeted for each sample.

Pre-processing and quality control -The 10x CellRanger 8.0 pipeline was used to perform read alignment and generate feature-barcode matrices, alongside quality control including introns (GRCm38/mm10). The *remove-backgroup function* in CellBender (v0.2.2) rescued RNA that was identified as empty droplets and removed empty droplets identified as RNA (ambient RNA) (12). Using the R package Seurat v5.0, filtered feature-barcode matrices were imported as Seurat objects and subsequent sample integration, count normalization, cell clustering, and marker gene identification was performed (13). Samples were filtered to remove low quality nuclei (defined as nuclei with < 200 or > 5000 genes or < 400 UMIs). Nuclei with > 5% reads from mitochondrial genes and/or > 5% reads from ribosomal protein genes were excluded from further analyses. Each sample had predicted doublets removed via DoubletFinder (14) and was normalized via *SCTransform* (15,16). Placental samples from the same timepoint (E11.5 or E17.5) were integrated using *SelectIntegrationFeatures*, *PrepSCTIntegration*, *FindIntegrationAnchors* and *IntegrateData* from the Seurat package. Both integrated datasets were subjected to unsupervised clustering using *FindNeighbors* (dimensions = 1:0) and *FindClusters* (resolution = 1.5 and 1.0 for E11.5 and E17.5 respectively) from the Seurat package.

Cell cluster annotation - Annotation was performed using the *FindMarkers* function in Seurat. Manual cell type assignments were conducted based on expression of reported markers (see *Results*, Fig. S6, Fig. S7, Dataset S8*A*, and Dataset S9*A*).

Pseudobulk analysis of differential gene expression - Genes that had low levels of expression (read count < 10) were filtered out before count aggregation across nuclei for a given cell cluster in a given biological sample. Reads from each sample were inputted into DESeq2 for differential gene expression analysis. The gene list was ranked by $-\log_{10}$(adjusted *P*-value)*$\log_2$(fold-difference) and GSEA was run on the ranked gene list set employing the fgsea R package (9). We used the orthology-mapped Hallmark gene sets and the Ontology gene sets from MSigDB (10); pathways were selected and ranked based on the normalized enrichment score, adjusted *P*-value, and the number of significant leading-edge genes.

Immunohistochemistry (IHC)
Paraffin-embedded sections were deparaffinized and antigen retrieval was performed by boiling in Tris-EDTA Buffer pH 9.0 buffer (Abcam, ab93684) for 20 minutes. Slides were then washed in Tris-buffered saline (TBS) with 0.025% Triton X-100, and blocked for 2 hours at room temperature with 10% normal goat serum (Abcam, ab7481) and 1% FBS in TBS. Primary antibodies were diluted in antibody diluent (Abcam, ab64211) according to Table S1 and incubated overnight at 4°C. After washing with TBS-Triton-X 100, sections were incubated with

secondary antibodies at a concentration of 2 mg/mL in TBS supplemented with 1% FBS. Sections were mounted with Fluoroshield Mounting Media containing DAPI (Abcam, #ab104139). Stained sections were scanned with a Hamamatsu NanoZoomer (HT) imager (CD31, vimentin, phos-VEGFR2, VEGF-A, and angiopoietin-2) or a 3DHistech P250 imager (phos-p38-MAPK, phos-ERK1/2, progesterone receptor, and cytokeratin 7). Histological analysis was performed in a blinded fashion, using QuPath for fluorescence quantification (17).

Flow cytometry of decidual tissue
Whole deciduas were dissected apart from the placenta and pooled from a dam's entire litter as a single sample. Using procedures adapted from a previously published report (18), decidual tissue was minced for 3 minutes in Accumax solution (Innovative Cell Technologies, #AM105), and incubated for 30 minutes at 37°C with shaking at 80 rpm. The sample was centrifuged at 500 x g for 5 minutes and subsequently at 1,500 x g for another 5 minutes at 4 °C. The cell pellet was then filtered through sterile filters with 70 mm and 40 mm pore diameters (Fisher Scientific, #08-771-2 and #08-771-1, respectively). Between each filtering step, cells were washed with sterile Dulbecco's PBS (DPBS) and recovered by centrifugation. Decidual cells were stained with anti-CD16/32 Fcɣ block (Biolegend, #101320). Dead cells were excluded using the Zombie Red fixable viability kit (Biolegend, #423109). Super Bright Complete Staining Buffer (eBiosciences, #SB-4401-75) was used during antibody-fluorochrome conjugate staining to reduce nonspecific polymer dye-dye interactions. Samples were analyzed using the Cytek Northern Lights spectral cytometer. The fluorophore-labeled monoclonal antibodies used for flow cytometry are listed in Table S2. Representative gates are shown in Fig. S1*E*. The data were analyzed using FlowJo v10.10.0.

Protein and steroid hormone assays of placental homogenates
Homogenates were generated by taking two-thirds by weight of an individual placenta and adding 1 mm diameter Zirconia/Silica beads (BioSpec, #11079110z) and 600 mL of cold T-PER buffer (Thermo Fisher Scientific, #78510) with Complete Ultra protease inhibitor (Roche) and then adding the mixture to a bead beater. Bead beating was performed for 1 minute at 21°C. Samples were then centrifuged at 13,000 x g for 5 minutes at 4 °C and protein concentrations were measured in Dulbecco's phosphate-buffered Saline (DPBS) using a Pierce BCA Protein Assay Kit (Thermo Fisher Scientific, #23225). The homogenate samples were adjusted to a concentration of 1 mg/mL using sterile PBS with protease inhibitor cocktail (Roche, #11697498001) and then analyzed by Eve Technologies Corporation (Calgary, Canada) using their Mouse Angiogenesis and Growth Factor 16-Plex Discovery Assay® Array (MDAG16) and Steroid/Thyroid 5-Plex Discovery Assay® Multi Species Array for non-blood based samples (STTHD-Cell/Tissue), respectively.

Supplementary References

1. B. Marsh, R. Blelloch, Single nuclei RNA-seq of mouse placental labyrinth development. *eLife* **9**, e60266 (2020).
2. A. C. Nelson, A. W. Mould, E. K. Bikoff, E. J. Robertson, Single-cell RNA-seq reveals cell type-specific transcriptional signatures at the maternal–foetal interface during pregnancy. *Nat Commun* **7**, 11414 (2016).
3. X. Jiang, *et al.*, A differentiation roadmap of murine placentation at single-cell resolution. *Cell Discov* **9**, 30 (2023).
4. Y. Alippe, *et al.*, Fetal MAVS and type I IFN signaling pathways control ZIKV infection in the placenta and maternal decidua. *J Exp Med* **221**, e20240694 (2024).
5. R. Y. Chen, *et al.*, A Microbiota-Directed Food Intervention for Undernourished Children. *N Engl J Med* **384**, 1517–1528 (2021).
6. B. J. Callahan, *et al.*, DADA2: High-resolution sample inference from Illumina amplicon data. *Nat Methods* **13**, 581–583 (2016).
7. H. Mallick, *et al.*, Multivariable association discovery in population-scale meta-omics studies. *PLOS Computational Biol* **17**, e1009442 (2021).
8. M. L. Love, W. Huber, S. Anders, Moderated estimation of fold change and dispersion for RNA-seq data with DESeq2. Genome Biol **15**, 550 (2014).
9. G. Korotkevich, *et al.*, Fast gene set enrichment analysis. *bioRxiv* (2021). https://doi.org/10.1101/060012. [Accessed 16 September 2024].
10. A. S. Castanza, *et al.*, Extending support for mouse data in the Molecular Signatures Database (MSigDB). *Nat Methods* **20**, 1619–1620 (2023).
11. H.-W. Chang, *et al.*, *Prevotella copri* and microbiota members mediate the beneficial effects of a therapeutic food for malnutrition. *Nat Microbiol* **9**, 922–937 (2024).
12. S. J. Fleming, *et al.*, Unsupervised removal of systematic background noise from droplet-based single-cell experiments using CellBender. *Nat Methods* **20**, 1323–1335 (2023).
13. Y. Hao, *et al.*, Dictionary learning for integrative, multimodal and scalable single-cell analysis. *Nat Biotechnol* **42**, 293–304 (2024).
14. C. S. McGinnis, L. M. Murrow, Z. J. Gartner, DoubletFinder: Doublet Detection in Single-Cell RNA Sequencing Data Using Artificial Nearest Neighbors. *Cell Syst* **8**, 329-337.e4 (2019).
15. C. Hafemeister, R. Satija, Normalization and variance stabilization of single-cell RNA-seq data using regularized negative binomial regression. *Genome Biology* **20**, 296 (2019).
16. S. Choudhary, R. Satija, Comparison and evaluation of statistical error models for scRNA-seq. *Genome Biol* **23**, 27 (2022).
17. P. Bankhead, *et al.*, QuPath: Open source software for digital pathology image analysis. *Sci Rep* **7**, 16878 (2017).
18. M. Arenas-Hernandez, E. N. Sanchez-Rodriguez, T. N. Mial, S. A. Robertson, N. Gomez-Lopez, Isolation of Leukocytes from the Murine Tissues at the Maternal-Fetal Interface. *J Vis Exp* **21**, e52866 (2015).
